# Implementation of risk stratification within bowel cancer screening: a community jury study exploring public acceptability and communication needs

**DOI:** 10.1186/s12889-023-16704-6

**Published:** 2023-09-15

**Authors:** Lily C. Taylor, Rebecca A. Dennison, Simon J. Griffin, Stephen D. John, Iris Lansdorp-Vogelaar, Chloe V. Thomas, Rae Thomas, Juliet A. Usher-Smith

**Affiliations:** 1 Primary Care Unit, Department of Public Health and Primary Care, School of Clinical Medicine, University of Cambridge, Cambridge, UK; 2 MRC Epidemiology Unit, School of Clinical Medicine, University of Cambridge, Cambridge, UK; 3 Department of History and Philosophy of Science, University of Cambridge, Cambridge, UK; 4 Department of Public Health, Erasmus University Medical Centre, Rotterdam, The Netherlands; 5 School of Health and Related Research, University of Sheffield, Sheffield, UK; 6 Department of Public Health and Tropical Medicine, James Cook University, Queensland, Australia

**Keywords:** Bowel cancer, Risk stratification, Cancer screening, Community jury, Acceptability

## Abstract

**Background:**

Population-based cancer screening programmes are shifting away from age and/or sex-based screening criteria towards a risk-stratified approach. Any such changes must be acceptable to the public and communicated effectively. We aimed to explore the social and ethical considerations of implementing risk stratification at three different stages of the bowel cancer screening programme and to understand public requirements for communication.

**Methods:**

We conducted two pairs of community juries, addressing risk stratification for screening eligibility or thresholds for referral to colonoscopy and screening interval. Using screening test results (where applicable), and lifestyle and genetic risk scores were suggested as potential stratification strategies. After being informed about the topic through a series of presentations and discussions including screening principles, ethical considerations and how risk stratification could be incorporated, participants deliberated over the research questions. They then reported their final verdicts on the acceptability of risk-stratified screening and what information should be shared about their preferred screening strategy. Transcripts were analysed using codebook thematic analysis.

**Results:**

Risk stratification of bowel cancer screening was acceptable to the informed public. Using data within the current system (age, sex and screening results) was considered an obvious next step and collecting additional data for lifestyle and/or genetic risk assessment was also preferable to age-based screening. Participants acknowledged benefits to individuals and health services, as well as articulating concerns for people with low cancer risk, potential public misconceptions and additional complexity for the system. The need for clear and effective communication about changes to the screening programme and individual risk feedback was highlighted, including making a distinction between information that should be shared with everyone by default and additional details that are available elsewhere.

**Conclusions:**

From the perspective of public acceptability, risk stratification using current data could be implemented immediately, ahead of more complex strategies. Collecting additional data for lifestyle and/or genetic risk assessment was also considered acceptable but the practicalities of collecting such data and how the programme would be communicated require careful consideration.

**Supplementary Information:**

The online version contains supplementary material available at 10.1186/s12889-023-16704-6.

## Background

Population-based screening programmes have been established in many countries to reduce bowel cancer incidence and mortality [1, 2]. The English bowel cancer screening programme currently invites men and women aged 56–74 to complete faecal immunochemical testing (FIT) with a two-year screening interval, although the starting age is gradually being reduced to age 50 [3]. Individuals with a FIT result above a given threshold (currently 120 µg/g) are offered a colonoscopy, with return to the screening population for those with negative findings [4].

The likelihood of being diagnosed with bowel cancer varies according to multiple individual-level risk factors such as age, sex, body mass index (BMI), faecal haemoglobin concentration at previous FIT screenings, lifestyle/phenotypic risk and genetics [5]. As a result, there are considerable variations in the absolute benefit that an individual may derive from bowel screening, with those at higher risk having a more favourable harm-benefit ratio than low-risk groups. Resource constraints, combined with concerns about the balance of these benefits and harms, has prompted a shift towards risk-stratified screening [6]. Within a stratified screening programme the age at first invitation, screening interval and/or FIT threshold for colonoscopy referral could be tailored according to individual risk. Using age, sex and FIT results represent an opportunity for risk stratification using data readily available within existing screening programmes that would incur little to no extra cost [7]. For example, incorporating stratification into the threshold for referral for further investigations could result in an individual who is at higher risk due to their age and sex being referred for colonoscopy with a lower concentration of blood on FIT. Such an approach could use the FIT concentration from the current screening round and/or prior results. Lifestyle and genetic risk factors could also be included but would necessitate additional investment for both participants and the healthcare system, such as undertaking genetic testing and completing questionnaires or measurement to record up to date lifestyle characteristics.

Risk prediction models based on age, sex, and FIT results have demonstrated the potential to identify those at higher risk of having a bowel cancer or polyp detected at colonoscopy (area under the receiver operator curve [AUC] 0.71), and models incorporating additional lifestyle data have good discriminatory ability (AUC 0.76) [5, 7]. At present, genetic risk prediction models have limited predictive capabilities but improvements associated with the identification of more genes related to bowel cancer risk and progression of machine learning techniques are anticipated [5].

The success of a risk-stratified bowel cancer screening programme is dependent on sufficient uptake and therefore on acceptability and comprehension from the perspective of participants and society as a whole [8]. Risk stratification raises issues of equity when considering systematic differences between population sub-groups and challenges social perceptions of the value of screening, particularly concerning de-escalation of screening for low-risk individuals [9–11]. Additionally, there are multiple possible points amenable to risk stratification within the bowel cancer screening pathway, as well as different variables that may contribute to risk modelling. There is, therefore, a need to understand public attitudes towards these different strategies and how best to communicate them.

Community juries are a democratic method in which participants are informed about the topic and are then asked to deliberate and reflect on their views before delivering a final verdict on the research question(s). This process encourages participants to think beyond their individual perspective to consider the views of wider society [12]. This method is particularly useful when applied to research questions that require consideration of values and evidence, such as the ethics of resource allocation [13, 14]. We used community juries to explore social and ethical considerations relating to using risk stratification at three points on the bowel cancer screening pathway. Secondarily, we sought to understand how best to communicate the preferred screening strategies to the wider public.

## Methods

### Study design

We conducted two pairs of community juries, reported using the ‘CJCheck Framework’, a reporting standards checklist developed to promote systematic and transparent reporting of community jury studies [15]. They explored the use of risk stratification at three points on the bowel cancer screening pathway that are amenable to risk stratification and apply to all participants [16]:

1. Eligibility: age at first invitation (juries 1 and 2)
2. FIT threshold: faecal haemoglobin concentration at which someone is referred for colonoscopy (juries 3 and 4)
3. Screening interval: frequency of screening (juries 3 and 4)

Information about the study design is available in greater detail within the study protocols (see Data Availability).

### Research team

The research team consisted of eight researchers across all community juries, including academic clinicians, public health researchers and researchers with expertise in community jury methods. Three patient and public involvement (PPI) representatives were involved in designing the protocol and participant facing information alongside two members of the research team (RD, LT). Four members of the research team, chosen for the relevance of their expertise in public health and/or risk-stratified screening, delivered jury presentations (SG, SJ, IL, CT).

### Participants

We purposefully recruited jury members by age, sex, ethnicity, social grade, geographic region and screening history using iPoint Research Ltd, a market recruitment company. In juries 1 and 2, participants were aged 40–74 years to reflect the likely screening population plus the age at which a risk assessment to determine eligibility would be conducted. Participants in juries 3 and 4 were aged 50–74 years as this is the current target age range for bowel cancer screening across England, Scotland and Wales. Participants were excluded if they had a personal history of bowel cancer, expertise in medicine or had previously participated in our related community jury [17]. iPoint Research allocated participants to the juries based on their individual availability and in a way that ensured a balance of demographic characteristics. iPoint Research also obtained informed consent, provided relevant organisational and study details and reimbursed participants at their recommended rate.

### Procedure

Each community jury involved two sessions of up to four hours duration, held over two consecutive days between February and May 2022. All juries took place online using Zoom videoconferencing software (Zoom Video Communications) to facilitate sampling according to geographical region. Participants received an information pack via email prior to the juries including a summary of previous jury findings, glossary of key terms and presenter slides and biographies (see Data Availability). We asked participants to complete a questionnaire before and after the juries, based on a previously developed questionnaire [18], to collect demographic information (pre-jury questionnaire only), and to compare individual attitudes to cancer screening before and after the juries using a six-point Likert scale (see Data Availability).

Participants first heard information from expert presenters with an opportunity to ask questions before being asked to discuss their views and provide a verdict on which options for risk stratification are acceptable and under what circumstances (Table 1). Jury members were asked to consider risk stratification from a community/societal perspective and assume that any changes to the screening programme would be supported by appropriately accurate and validated risk prediction models. Suggested approaches to risk stratification of the eligibility criteria included using lifestyle factors (including age, sex, ethnicity, BMI, smoking, drinking and family history) and genetic risk. Suggested approaches to risk stratification of FIT thresholds and screening intervals discussed the use of age, sex and FIT results as currently available data and subsequently introduced future perspectives on risk stratification using lifestyle and/or genetic factors. Expert videos (Table S1) were 15–20 min long and pre-recorded, and experts were available immediately after viewing for live Q&A sessions*.*

**Table 1:** Schedule for the community juries

Jury 1 and 2	Jury 3 and 4
Day 1	
Individual introductions and technology check	Individual introductions and technology check
Welcome, introduction and plan for the jury	Welcome, introduction and plan for the jury
Expert presentation 1: Why we screen for bowel cancer and what are the potential benefits and harms	Expert presentation 1: Why we screen for bowel cancer and what are the potential benefits and harms
Q&A 1	Q&A 1
Break	Break
Expert presentation 2: Ethical considerations around bowel cancer screening programmes	Expert presentation 2: Ethical considerations around bowel cancer screening programmes
Q&A 2	Q&A 2
Break	Break
Expert presentation 3: The potential effects of introducing risk stratification to determine entry into the bowel cancer screening programme	Expert presentation 3: The current approach to bowel cancer screening and how risk stratification could be incorporated (using age, sex, and FIT result)
Q&A 3	Q&A 3
Summary and end of day 1	Facilitated deliberation 1
	Summary and end of day 1
Day 2	
Welcome, plan for the day and reflections on day 1	Welcome, plan for the day and reflections on day 1
Facilitated discussion	Expert presentation 4: How risk stratification could be incorporated into bowel cancer screening in the future (using lifestyle and genetic factors)
Break	Q&A 4
Unfacilitated deliberation 1	Break
Present recommendations to senior author 1	Facilitated deliberation 2
Break	Break
Unfacilitated deliberation 2	Unfacilitated deliberation
Present recommendations to senior author 2	Present recommendations to senior author
Completion of questionnaires, summary, and end of day 2	Completion of questionnaires, summary, and end of day 2

Jury members were invited to discuss the information delivered by the experts and consider scenarios exemplifying risk stratification strategies during deliberation sessions that were co-facilitated by two researchers (RD, LT) and observed by at least one PPI member. Participants were then asked to engage in unfacilitated deliberations in order to reach a verdict on our research questions (Table 2). They nominated a spokesperson to act as head juror and provide their final feedback to the senior author (JUS). During this time the facilitators turned their camera and microphones off but were available via the Zoom chat. All facilitators and expert presenters remained impartial throughout the juries to avoid biasing jury members. All Q&A sessions, facilitated and unfacilitated deliberations and verdicts were recorded using Zoom and participants completed a final questionnaire at the close of the juries to compare their individual views before and after the study.

**Table 2:** Questions for unfacilitated deliberations in the community juries

Jury 1 and 2	Jury 3 and 4
Acceptability of risk stratification	
• How should we decide *who to invite* for bowel cancer screening and why? • Any conditions/ caveats?	• Is it acceptable to use information we currently have available (age, sex, previous screening result) to decide *the cut-off for a positive FIT test* and why? • Is it acceptable to use information we currently have available (age, sex, previous screening result) to decide *screening intervals* and why? • Are there any requirements that would need to be met in order for this to be acceptable?
	• Is it acceptable to use other types of information you have heard about (lifestyle and genetics) to decide *the cut-off for a positive FIT test* and why? • Is it acceptable to use the other types of information you have heard about (lifestyle and genetics) to decide *screening intervals* and why? • Are there any requirements that would need to be met in order for this to be acceptable?
Communication preferences	
• How should we communicate this to the public? (Jury 1) • What information does the public need to know about your screening strategy? (Jury 2) • What information would you include on the bowel cancer screening website?	• What information does the public need to know about these screening strategies? • What information would you include on the bowel cancer screening website/leaflet?

### Analysis

Study recordings were externally transcribed verbatim and pseudonymised. We then analysed them using codebook thematic analysis [19], led by RD and LT, as there were clearly defined questions and topics for discussion within the jury structure. We familiarised ourselves with the data by reviewing transcripts, audio recordings and field notes, including personal reflections, and used these to independently generate an initial high-level coding frame (Table S2), which was refined during consensus meetings. Transcripts were coded according to this framework using NVivo 12 (QSR International). This process was repeated using the data within the initial codes to develop a framework for coding sub-themes. Data were aggregated and unifying themes were established. This was an iterative process and researchers continued to review the coded data and refine any identified themes via consensus meetings.

Quantitative data from the pre- and post-jury questionnaires were analysed using Stata 15. Wilcoxon signed rank tests were used to identify changes in individual attitudes towards screening and risk stratification before and after the community juries.

## Results

### Participants’ characteristics and individual views on risk stratification

A total of 31 participants took part across four juries. As shown in Table 3, a range of characteristics and demographics were included in each jury. Twenty-one participants (67.7%) did not have a university degree and eight (25.8%) were working class (social grade C2DE). Two participants in juries 3 and 4 (6.4%) had previously been invited to participate in cancer screening but had chosen not to attend. Their beliefs about cancer are summarised in Figure S1.

**Table 3:** Participants’ demographic characteristics

	**Jury 1**	**Jury 2**	**Jury 3**	**Jury 4**	**All (%)**
Total N	7	8	7	9	31 (100.0)
Age range					
40–49 years	1	3	0	0	4 (12.9)
50–59 years	2	2	3	2	9 (29.0)
60–69 years	2	3	3	5	13 (41.9)
70–74 years	2	0	1	2	5 (16.1)
Sex					
Male	4	4	3	5	16 (51.6)
Female	3	4	4	4	15 (48.4)
Ethnicity					
White	5	6	6	7	24 (77.4)
Mixed/multiple ethnic group	0	0	0	1	1 (3.2)
Asian/Asian British	1	2	1	0	4 (12.9)
Black, African, Caribbean/ Black British	1	0	0	1	2 (6.5)
Education					
Not completed A levels or equivalent	1	4	2	2	9 (29.0)
Completed A levels or equivalent	1	2	2	2	7 (22.6)
Completed further education but not a degree	1	1	1	2	5 (16.1)
Completed a Bachelor's degree or above	4	1	2	3	10 (32.3)
UK region					
England (London and South East)	4	3	3	5	15 (48.4)
England (North or North West)	2	0	2	4	8 (25.8)
England (Midlands)	1	2	1	0	4 (12.9)
England (Yorkshire and the Humber)	0	2	1	0	3 (9.7)
Wales	0	1	0	0	1 (3.2)
Social grade					
B (middle middle class)	1	3	3	2	9 (29.0)
C1 (lower middle class)	3	3	2	6	14 (45.2)
C2 (skilled working class)	2	0	2	1	5 (16.1)
D or E (working class or retired)	1	2	0	0	3 (9.7)
Self-reported health					
Excellent	3	0	0	1	4 (12.9)
Very good	1	2	2	2	7 (22.6)
Good	3	3	2	4	12 (38.7)
Fair	0	2	3	1	6 (19.4)
Poor	0	1	0	1	2 (6.5)
Smoking status					
Never smoke cigarettes or cigars	6	4	3	3	16 (51.6)
Used to smoke cigarettes or cigars	1	3	2	4	10 (32.3)
Smoke up to 20 cigarettes or cigars per day	0	1	2	2	5 (16.1)
Self-reported weight					
About the right weight	3	2	1	5	11 (35.5)
Slightly overweight	3	3	4	4	14 (45.2)
Very overweight	1	3	2	0	6 (19.4)

Individually, participants tended to be comfortable with different elements of risk-stratified screening eligibility, or consider them reasonable, in questionnaires conducted both before and after the juries with the modal response being ‘very’ comfortable/reasonable (juries 1 and 2; Figure S2). Statistically, participants were slightly more comfortable with delaying screening if found to be low risk according to the lifestyle risk score and had a more favourable perception of providing a sample for genetic testing after the juries. Similarly, most participants found the different elements of risk-stratified screening intervals and FIT thresholds to be very acceptable before and after the juries (juries 3 and 4; Figure S3). Small, statistically significant increases in this measure were observed for acceptability of using individual risk, sex, age and screening result to determine FIT cut-offs and/or screening intervals.

**Fig. 1:**
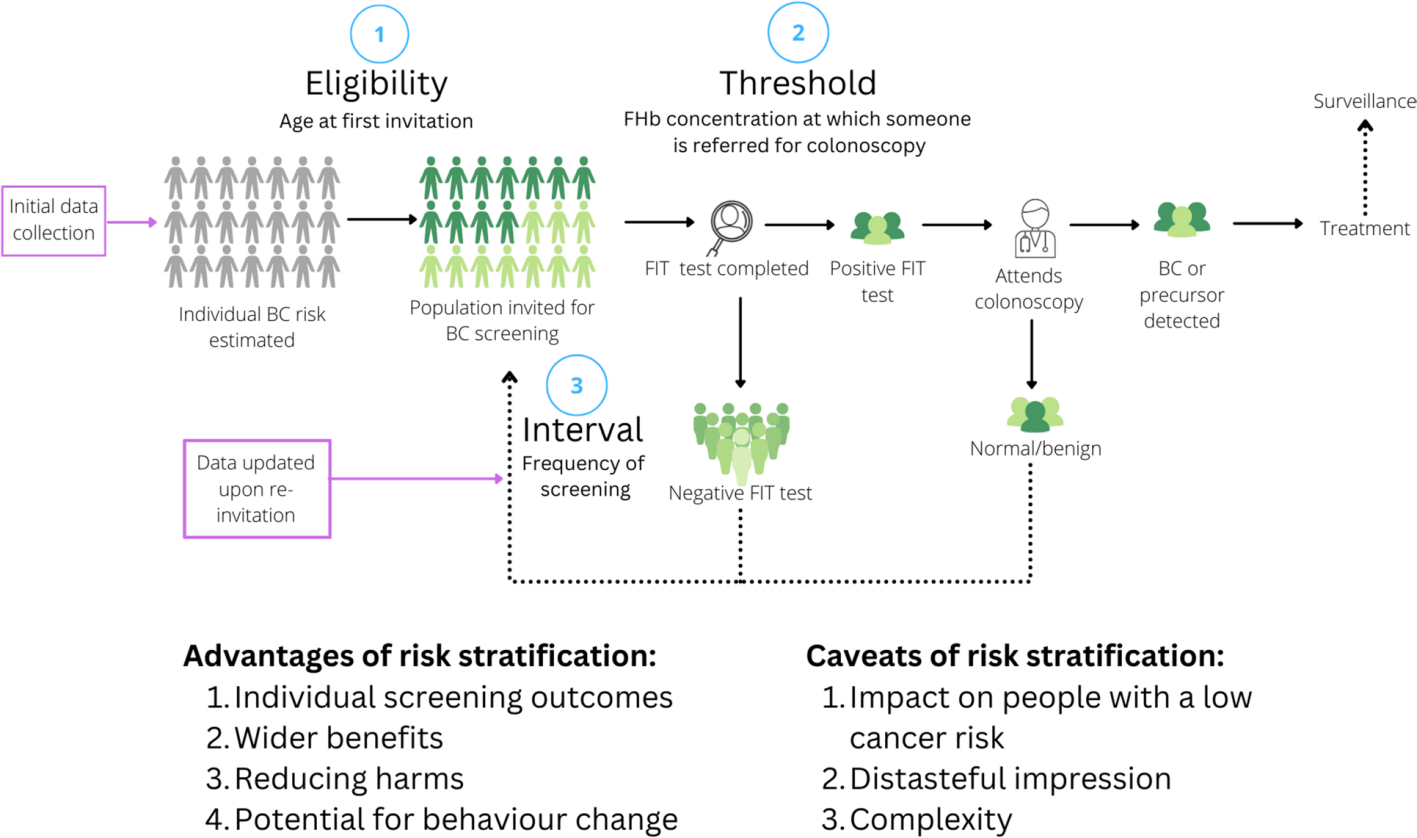
Opportunities for implementation and data collection across the bowel cancer screening pathway including advantages and caveats of risk stratification. BC Bowel cancer. BMI Body mass index. FIT Faecal immunochemical test

Jury discussions and verdicts about risk-stratified bowel cancer screening are presented in the following sections and summarised in Fig. 1. Prominent advantages, concerns and communication preferences tended to apply equally across the three points in the screening pathway so combined results from all four juries are presented. The final verdicts are reported separately for eligibility criteria, screening intervals and FIT thresholds. Supporting quotations are presented in Table 4.

**Table 4:** Participant quotations according to theme

**(A) Advantages of risk stratification**
1. “[…] finding, tweaking and toggling to a point where it works to the benefit of the NHS, to the benefit of the public, like what are the things that we can do that’s a little bit better that drives a greater result, and it presents to me a form of sort of joined-up thinking that we don’t always see in our approach to medical solutions.” – P1.4
2. “[…] those that are highest as risk will get the immediate care that they need rather than putting resources where they are not needed in the short term.” – P3.2
3. “If there was a choice out of a 20 year old getting screened and a choice out of me, say, at 70 getting screened I’d rather the 20 year old, in my personal opinion, because they’ve still got their life, I’ve had mine.” – P2.5
4. “[…] then you’ve got a better use of NHS resources so there’s probably less wastage with time, money and staff, that kind of thing, equipment, and therefore anything that’s saved can be better used in other areas of the NHS.” – P2.3
5. “It’s scary but I would rather, if I am at high risk, be at the total top of the priority list […] and really making sure that they are taken through a journey, that the funds are available and everything’s available to… I know we’re assuming funds are all there but at least then the focus is on me completely rather than, you know, willy-nilly worrying about everybody that’s potentially never going to get it.” – P3.2
6. “[…] you’re not necessarily doing it for their benefit, you’re doing it for the benefit of the amount of money you’re spending in the NHS. If you don’t sort people like that out, right, and they get missed it’s going to cost the NHS a lot more money further on down the line. So it’s not just about ‘Oh, well those people don’t look after themselves so we’re not looking after them’, it’s a case of ‘Well, okay, those people don’t look after themselves, but we need to make sure that we take the appropriate action to stop them costing us a hell of a lot more money.’” – P4.7
7. “I think in terms of the false positives, I think the sentiment might be… to be brutally honest, maybe we thought that was almost collateral damage for the greater good, unfortunately.” – P1.4
8. “And [the high-risk label] might prompt people to make lifestyle changes over the long term, you know, because it keeps it fresh in their mind that, yeah, maybe I don’t want to be going for the screening quite so often, so long term they might make more positive changes.” – P4.5
**(B) Caveats of risk stratification**
1. “We wouldn’t like to see lower risk people being punished for higher risk attending earlier, so essentially wanting the best of both worlds. We’d like early intervention but that shouldn’t be to the detriment of the lower risk and they should still get their screening on time, so to speak.” – P1.4
2. “It’s a very difficult one but my first instinct is that if they both have the same blood count then they do deserve the same place in the queue.” – P4.6
3. “…there’s so many different variables, you know, culture, lifestyle and so on, and you could model it on all of these variables but that probably is a very long term thing because it’s incredibly complex and then how do you then assess the kind of treatments or what’s the next step for those various models, you know, because you’d have twenty, thirty, fifty different variables.” – P3.2
4. “I’d want a condition in place that it doesn’t burden the NHS and take away from other vital services and resources, doesn’t infringe on anything else, because then… even just with cancer alone there’s two hundred others so I wouldn’t want to be taking away from one to give to the other because then that’s fair but then how do we quantify that?” – P2.3
**(C) Acceptability of risk-stratified bowel cancer screening**
1. “So maybe at 20 [years old] you might only have 1 point for your age but if you’ve got a family history you’re going to have a 10 on that one. Everybody is in the scale at some point.” – P2.3
2. “I think that’s important if there’s data to support the fact that there is more likelihood in certain ethnic groups that bowel cancer is prevalent but, again, I would like there to be some hard evidence.” – P1.6
3. “I think [BMI is] probably a better indicator than perhaps giving out a questionnaire from a GP or that, you know, to actually ask about your lifestyle. I think initially it’s probably, as P1.4 said, a better indicator of perhaps where you are sort of healthy lifestyle-wise.” – P1.6
4. “I don’t think lifestyle factors should be included because… You know, if people have an odd drink, fine, but if people end up being an alcoholic obviously there’s things out there to help them, you know, meetings and various other things, but I don’t think you can include somebody’s own personal choice, a lifestyle choice, even though they might be at higher risk in getting something. I don’t think it’s fair on other people, basically. It means that they’ll get tested against people that decided not to be a heavy drinker, not to smoke, and I just feel that that would be unfair.” – P2.7
5. “And I think that people mostly accept that there are differences between men and women in life, you know, things happen at different ages for all sorts of people, things affect men that don’t affect women and vice versa, so I don’t really see it being a big issue […] so long as they were furnished with the facts about why this was being done.” – P3.1
6. “It might not be best for everyone but would hopefully be the best for most people.” – P4.6
7. “I think they should all be included; I think it’s necessary. All the facts in front of you help… can’t not help actually.” – P3.4
8. “…Genetics, yes, which we all say is a factor that needs to be included, I think we all said this morning, but it’s not sort of immediate. The first one [age, sex and FIT result] was sort of like this can happen now, it’s the low-hanging fruit, and then moving on to the long term thing which was genetics and lifestyle and so on.” – P3.2
9. “I think there was a view expressed that lifestyle should not be mandated or sort of… it should not be an issue for discrimination for not having treatment and that any sort of questionnaire it would be up to the patient to decide what personal information the person would reveal about their lifestyle, they could not be forced to do it.” – P4.6
10. “As at the moment with the Covid, it’s the clinically extremely vulnerable are the top of the risk factor, it’s sort of I suppose weighting in statistics those items, whether it’s exercise, whether it’s your family, your diet, you know, whether you’re obese, you know, your BMI. That’s down, as I say, to doctors with the figures…” – P2.8
**(D) Communication preferences**
1. “Obviously we’d want people to know why they’re screened, what test is used, and why and who would be eligible. We wanted to have the risk factors on there so people could see who’s more susceptible, so we want to put in some like statistics and diagrams so you could see in simple language that this person is more likely to get it and why.” – P2.3
2. “I wouldn’t like to receive a letter that says I’m low risk, medium risk or high risk. Low risk would make me a bit blasé and just not bothered. High risk would make me panic. So I’d just prefer to say, “You’re going to be invited again in two years” or whatever it is, that would be fine. And you wouldn’t be comparing with like other people and just go like, “Well what risk did you get?” it’s just you just go when… It’s not different to me than going to the dentist, do you know what I mean, like some people might go every six months, some might need to go every year, so it’s just kind of quite matter of fact.” – P4.5
3. “I would want to know if I was high risk. I would want to know because it would ensure that I would make sure I send the test back.” – P3.7
4. “Those people who are worried about finding out their risk category, would you then prefer to get a letter that said that ‘You need to have a test. We can tell you what category you’re in but that’s up to you to come back to us to ask us?’” – P4.7
5. “We looked at actually cancer preventative advice, whether that’s specific for bowel cancer or just cancer in general, and we thought that might be a good thing to put on the website, because if people are looking on there anyway for information […] then they’re arming themselves with information that they can help, and that links in I guess with the lifestyle choices so they’re educating themselves” – P2.3
**(E) Reflections**
1. “…there’s obviously something in the 62 year old man that needs investigating and which age or anything shouldn’t come into it if there’s a possibility and it’s really, really important catching it in the early stages.” – P3.4
2. “I know that men are disproportionately affected and white males are affected more but then if you exclude all other ethnicities and if you exclude all women then I think that might be grossly unjust.” – P2.3
3. “I think smokers pay a lot of tax so they should be entitled to be screened and treated. I think smokers are probably propping up the NHS so they shouldn’t be denied any treatment or any tests.” – P2.4

*BMI* Body mass index, *NHS* National health service

### Advantages of risk stratification

Many benefits of a risk-stratified bowel cancer screening programme were salient to the participants. One juror remarked that this approach represents an example of “joined-up thinking” (Participant [P]1.4) with a diverse range of anticipated benefits (Table 4, Quote-A1).

#### Individual screening outcomes

Risk stratification was viewed as a way for individuals to benefit, since “the people at the highest risk, for definite, are the ones that need to take priority” (P2.7). Jurors were glad that risk stratification could facilitate high-risk people to access increased support and resources in a timelier manner (Table 4, Quote-A2), as well as enable their screening at a younger age since they wanted screening to begin earlier for everyone, appreciating that younger people have more to gain (Table 4, Quote-A3). The benefits of a better prognosis with early detection were also emphasised.

#### Wider benefits

Beyond individual benefits, advantages of risk stratification included increased cost-effectiveness and optimisation of healthcare resources, such as time, money and staff (Table 4, Quote-A4). The financial advantages were remarked upon by jurors in the context of a financially constrained health system and in relation to potential opportunity costs. Participants considered how money saved from risk stratification could be used efficiently to benefit other areas within the NHS, such as cancer treatment and services for other conditions: “I wouldn’t want to be tested; if I’m at low risk just leave me alone and let others get on with it… take my £6 [FIT kit] and spend it somewhere else” (P3.2). Participants in jury 3 highlighted the potential to adequately support high-risk individuals throughout screening and beyond, in terms of diagnosis and treatment, by redistributing resources and funding effectively (Table 4, Quote-A5).

Other participants raised the idea that screening unhealthy people who are high risk as a result of poor lifestyle choices is ultimately worthwhile in order to benefit the health service. In this situation, they were motivated by saving money and resources in the long-term rather than benefits to such individuals (Table 4, Quote-A6).

#### Reducing harms

Several jurors commented that the harms of screening were more serious than they had previously thought, in particular the potential for emotional and psychological harm. However, discussion tended to focus more on the benefits of risk stratification for high-risk people who may receive intensified screening, rather than the benefits for low-risk people who may avoid harms (Table 4, Quote-A7). Nevertheless, participants did note the potential to avoid the negative psychological impacts of unnecessary screening and overtreatment for people who are at low risk.

#### Potential for behaviour change

A final benefit of risk stratification was the potential for behaviour change. Improved awareness of risk factors as a result of the programme as well as discovering their individual bowel cancer risk was anticipated to motivate some to adopt healthier “principles” that “you actually do know […] that you ought to be following” (P1.6), particularly if they had a high risk (Table 4, Quote-A8).

### Caveats of risk stratification

Participants across all four juries considered the possible limitations of risk stratification.

#### Impact on people with a low cancer risk

Participants understood that risk prediction would not be perfect. Although only one participant was concerned about a high-risk label leading to overtreatment, the impact of risk stratification on individuals who are at low risk of developing bowel cancer was a pertinent issue. Some participants were pessimistic about the prospect of no screening for such groups and reduced screening was discussed as being unfair and even punitive (Table 4, Quote-B1).

##### Distasteful impression

Despite acknowledging the benefits, participants were also concerned that certain aspects (such as rationing) were, or could be perceived as, distasteful, or discriminatory. Participants in jury 1 considered the appearance of more screening for certain groups of society, specifically the potential for increased screening for white people versus other ethnicities to appear distasteful if ethnicity were used as part of a risk model. Participants in both juries 3 and 4 were concerned that risk-stratified FIT thresholds equated to rationing screening resources, particularly when presented with scenarios illustrating the implications on an individual basis in which participants felt uncomfortable making rationing decisions. As explained below, confusion that people with a non-zero level of blood in their faeces may be excluded from receiving a colonoscopy clearly contributed to this concern (Table 4, Quote-B2).

#### Complexity

Finally, participants questioned whether risk stratification might actually increase healthcare costs and workload rather than alleviating these issues. They speculated that the collection of additional data could be very time-consuming, particularly if numerous variables were needed for risk prediction, and some jurors even suggested that this may “overload the system” (P1.2) in terms of data collection, storage, and risk modelling (Table 4, Quote-B3). Participants considered how other NHS services, including other cancer screening programmes, might be impacted if risk stratification resulted in more screening and colonoscopies (Table 4, Quote-B4).

### Verdict: acceptability of risk-stratified bowel cancer screening

Following discussion of the advantages and caveats described above, all juries reached a consensus on the acceptability of risk-stratified bowel cancer screening.

#### Eligibility (point 1)

Juries 1 and 2 favoured risk-stratified bowel cancer screening over the current age-based eligibility, describing it as a “more targeted” and “multipronged” approach (P2.3) with a “sliding scale” (P1.2/4/5/6 and 2.3). Although screening would be offered differentially, this approach was considered fair because individuals across all demographics would be included within the risk assessment.

Akin to a risk prediction model, both juries explained how they would like to see different risk factors contributing more or less to inform the age at which someone was first invited based on relative risk and acceptability (Table 4, Quote-C1). As such, evidence of a factor’s significant contribution to bowel cancer risk was important (Table 4, Quote-C2). They were more certain about and/or placed higher priority on non-modifiable risk factors since the more contentious personal lifestyle choices would be avoided. As a result, risk stratification based on age, sex, ethnicity, family history and genetics was preferred. Furthermore, although it had been excluded earlier in the discussions, BMI was ultimately included because participants felt that it would give some indication of general health in a way that would be more acceptable than directly asking people about their lifestyle (particularly alcohol consumption and smoking) (Table 4, Quote-C3).

Although participants noted the associations with bowel cancer risk, the juries concluded that personal lifestyle choices should be discarded for several reasons. In terms of collecting the data, they expressed concerns that the public would not complete lifestyle questionnaires and that it would be inaccurate if they did because people “might embroider the truth” (P1.6) about their behaviours, either positively to appear healthier or negatively to access screening, or just find it too difficult to report behaviours that vary day-to-day. Additionally, many felt that people should be free to make lifestyle decisions without affecting their access to screening (Table 4, Quote-C4) and acknowledged that some people have greater potential to make healthy choices than others due to finances, age, and wider health.

#### FIT thresholds (point 2) and screening intervals (point 3)

Juries 3 and 4 viewed the concepts of FIT thresholds and screening intervals in the same way and did not find them to be fundamentally different in terms of acceptability. Their views on risk stratification at these points on the screening pathway are therefore reported together.

##### Using data within the system

Participants were unanimous in their decisions that using age, sex and FIT result is an acceptable strategy for risk stratification of FIT thresholds and screening intervals. They felt that using these factors would be a logical starting point as the data are already available, describing it as “low-hanging fruit” (P3.6), a “no brainer” (P4.3 and 4.7) or “an easy win” (P3.2). One participant likened stratification to other situations in life that may be determined by age or sex, suggesting that people are already familiar with such a concept and are therefore likely to find it acceptable (Table 4, Quote-C5). In jury 4 specifically, participants felt that FIT result should be weighted most heavily when calculating individual risk as blood in the stool “is a red flag irrespective of your age and your sex” (P4.6). However, they also acknowledged that a screening programme is unlikely to be a perfect fit for everyone (Table 4, Quote-C6).

##### Collecting additional data

When asked about the acceptability of collecting additional data to inform modelling for risk stratification beyond eligibility (points 2 and 3), participants were generally optimistic. Unlike in juries 1 and 2, participants had few reservations about lifestyle risk factors and largely believed that the more data included in the risk models the better (Table 4, Quote-C7).

However, participants felt that the addition of lifestyle or genetic information was a consideration for the future (Table 4, Quote-C8). This related to data collection and the potential for ethical challenges, such as data security, privacy and discrimination as described above.

As for the first two juries, a weighting system was suggested for each of the risk factors included in the risk model and participants emphasised that providing additional data should be a matter of personal choice, rather than being mandated (Table 4, Quote-C9). Jurors compared risk stratification with the Covid-19 vaccine rollout where high risk or clinically vulnerable individuals were prioritised and suggested a similar weighting system could be used to assess priority for bowel cancer screening (Table 4, Quote-C10).

### Communication preferences

#### Information about the programme

If risk stratification was implemented, participants would want to know details of the main risk factors for developing bowel cancer and statistics to support their use in determining screening programme features (Table 4, Quote-D1). However, several participants noted that there is potential to ‘scare people off’ by providing too much information, particularly about colonoscopies. Similarly, participants did not think the public needs to know how accurate risk modelling is (that should be “[left] up to the professionals” (P2.6)) and felt that communications should not be framed in the context of limited colonoscopies. While simple information could be provided in the screening letter, the option of being directed to supplementary information available online was suggested. This would enable individuals to learn more about the risk-stratified screening programme if they wanted, but others would not see it by default.

#### Information on personal risk

Desire for feedback about personal risk varied, with quite strongly held views. Some participants felt that knowing their risk was high could induce anxiety, or that knowing it was low might lead to complacency about lifestyle and screening decisions (Table 4, Quote-D2). Others would rather know their risk and how that had affected their screening schedule and advocated for full transparency: “I think the patient needs all the information they can get in order to understand the reasoning behind it” (P4.3). Discovering they were high risk could make some more likely to complete FIT testing (Table 4, Quote-D3). A possible solution suggested by participants to address these varying preferences was to stipulate an individual’s next screening date in their screening letter and to hold more detailed information about personal risk estimates within electronic medical records that they could easily request (Table 4, Quote-D4).

#### Language and communication channels

Throughout these discussions jury members emphasised the importance of positive, simple and lay-friendly language in public communication. They felt that using diagrams like those presented in the juries made information more digestible. They suggested many different channels and outlets for disseminating this information including television, radio, social media, community organisations, charities and GP surgeries. Participants discussed the accessibility of different forms of communication and suggested a tailored approach for different population groups, e.g. using charities and community groups to target older people who may not have internet access or use social media.

#### Wider awareness

A final consideration that came up frequently across all the juries is the perceived importance of public education to increase societal awareness of bowel cancer and screening, and preventive advice. Screening, particularly the initial invite, was often seen as a teachable moment and an opportunity to increase public awareness of modifiable risk factors (Table 4, Quote-D5).

### Reflections

#### Participants’ evaluation

Participants reported positive experiences of the juries, with the median response of ‘strongly agree’ across the four domains (presentations, facilitators, discussions, and outcomes) in the evaluation questionnaire (Figure S4).

#### Authors’ reflections on areas of confusion

Jurors learnt a lot of new information about the topic, which we endeavoured to present as clearly as possible, and they helped one another to understand it, yet they appeared to find certain aspects of risk stratification confusing. Some participants had concerns that people with blood in their FIT test would be excluded from receiving a colonoscopy on the basis of being low risk according to other factors, despite discussing this aspect with the experts (Table 4, Quote-E1). Generally, participants found the concepts of how eligibility and screening intervals could be stratified easier to grasp than FIT thresholds.

Additionally, there was some misunderstanding about the difference between similar concepts. For example, having a risk assessment and receiving screening and the distinction between family history and genetics. In jury 2 specifically, some participants mistakenly believed that certain behaviours may automatically qualify or exclude people from screening instead of contributing to the risk assessment (Table 4, Quote-E2/E3).

## Discussion

Incorporating risk stratification at distinct points on the bowel cancer screening pathway was acceptable to informed members of the public. Collectively, participants were convinced that the potential benefits gained by individuals and society justified moving away from age-based screening. Perceived benefits outweighed concerns that risk stratification would negatively impact people with an estimated low cancer risk, be considered unethical or be too complex. Clear and effective communication about the bowel cancer screening programme in general, but particularly the risk-stratified elements was, therefore, crucial.

Stratifying FIT thresholds and screening intervals based on age, sex and FIT levels was considered an obvious change to the current UK screening programme. Participants felt this could be implemented as soon as validated risk models and screening infrastructure are in place and would receive backing from society. In contrast with the later stages of the screening pathway (points 2 and 3), introducing risk stratification for eligibility (point 1) would necessitate collecting additional data about individuals. Participants decided that some risk factors were more acceptable to include here than others: they favoured age, sex, ethnicity, family history, BMI and genetics. Since the predictive ability of lifestyle risk was acknowledged in juries 1 and 2 and deemed more acceptable in juries 3 and 4, including these factors throughout the pathway has the potential to be satisfactory if their inclusion is thoroughly justified, and simple and accurate data collection is ensured.

If risk-stratified bowel cancer screening were implemented, it is clear that the public would want information to be available to enable them to understand what changes had been made and why, particularly how the included characteristics impact risk and how they could improve their individual risk level. Receiving feedback on personal bowel cancer risk was important but was preferred to be optional rather than automatic. Future research should focus on how to feasibly facilitate public understanding of these concepts, since the degree of education in a research context (particularly in community juries) is not possible at the population level. This could be achieved by co-designing and evaluating communication to be used in mass media campaigns and/or screening programme documentation, focusing on comprehension, acceptability and impact on intention for screening. Furthermore, the practicalities and effectiveness of providing some information and feedback individually (such as in the screening letter) with options to access more details elsewhere (such as online) must be evaluated. However, this is likely to become more feasible as more healthcare systems offer online interfaces and resources [20]. Additionally, further evidence is required for developing and validating risk prediction models, as well as research with the public to identify how best to group the population and to establish appropriate terms to describe those risk groups.

### Comparison with other literature

The general acceptability of risk stratification reported in this study aligns with previous research considering risk stratification across multiple cancer types among less informed participants [9, 17, 20–24]. Findings are also consistent with those from a similarly informed group of participants in our recent community jury study considering the use of risk stratification to determine eligibility for cancer screening in general [17]. Additionally, the high acceptability of providing personal information for cancer risk assessment reported here is consistent with results from previous studies in which many members of the public considered completing a questionnaire or providing a sample for genetic analysis acceptable for risk estimation [17, 24–27]. Participants in our study generally felt that the more information included in risk estimation the better; this is supported by a survey about the acceptability of different approaches to determining eligibility, where a comprehensive lifestyle risk score was preferred over less comprehensive models [18]. However, in line with findings from our previous community jury, participants had outstanding concerns about the inclusion of lifestyle data in risk modelling, with participants from both studies expressing a preference for non-modifiable lifestyle risk factors over modifiable ones [17]. Furthermore, focus groups on perceptions of risk-based breast cancer screening found that women felt strongly that participation in cancer risk assessment should be optional, which is congruent with the beliefs of jury participants [28]. Jury participants were more specific in their reasoning, stating the potential for gaming the risk assessment process or difficulty in accurately recalling habitual behaviours.

As in our study, the desire for clear and transparent public communication in relation to risk factors and changes to cancer screening programmes has been exemplified across different cancer types [21, 24, 28–30]. Additionally, a preference for diagrams and lay-friendly language when communicating risk information is well-evidenced [30–32]. In the context of recent changes to NHS England cervical cancer screening intervals, inadequate public communication has been demonstrated as a barrier to acceptability [33–35]. Jury participants suggested the use of multiple channels and outlets to communicate changes to the screening programme, the value of which is emphasised by low acceptability of extended cervical screening intervals after changes were implemented without public announcement [33].

### Strengths and limitations

The community jury method used in this study enabled us to gain an in-depth, society-centric understanding of the public’s views on risk-stratified bowel cancer screening and priorities for communication. Importantly, participants were informed about the topic, had opportunities to question experts and had time to contemplate their personal views and preferences for wider society. Although they found the topic complex, significant misunderstandings were addressed by the facilitators and the participants confirmed whether their views changed as result. Therefore these areas of confusion are unlikely to have affected participants’ overall conclusions about risk stratification, but this does highlight areas that the public may require more clarity on. The jury process was hypothetical in nature, and we were not able to, for example, elicit participants’ real life response if they were offered less frequent screening following a low bowel cancer risk estimate. Nonetheless, all acknowledged the seriousness of the topic and considered wide-ranging implications. While the facilitators remained impartial throughout and sought to encourage participants to express any divergent views that they held, they may still have held back from doing so.

An inevitable limitation is recruitment bias, specifically that we included individuals with more interest in cancer and screening than the general population. Overall, the participants viewed screening favourably, although the sample included some participants who had declined the invitation to attended bowel cancer screening. Using the recruitment agency is likely to have mitigated this somewhat compared to approaches such as advertising via posters in general practices. Our recruitment strategy and the online design enabled us to include participants from across the UK with a variety of demographic characteristics.

## Conclusions

Risk stratification of eligibility criteria, FIT threshold and/or screening intervals for bowel cancer screening was considered preferable to the current screening programme. Risk stratification using readily available data (age, sex and FIT result) was perceived favourably by the public and could be implemented immediately from the perspective of acceptability. The inclusion of additional lifestyle or genetic risk factors is acceptable but was considered aspirational due to the practicalities associated with data collection. Non-modifiable risk factors, such as age, sex, ethnicity and family history, may be more acceptable than modifiable risk factors. Additionally, some members of the public have a strong desire for information and future research should consider how best to communicate this.

### Supplementary Information

**Additional file 1: Supplementary Table 1.** Overview of the expert presentations included in the community juries. **Supplementary Table 2.** Thematic analysis coding frame. **Supplementary Table 3.** Pre-jury questionnaire (juries 1 and 2). **Supplementary Table 4.** Post-jury questionnaire (juries 1 and 2). **Supplementary Table 5.** Pre-jury questionnaire (juries 3 and 4). **Supplementary Table 6.** Post-jury questionnaire (juries 3 and 4). **Supplementary Figure 1.** Participants’ beliefs about cancer (collected in questionnaire 1). **Supplementary Figure 2.** Change in how reasonable or comfortable participants were with elements of risk-stratified bowel cancer screening eligibility (juries 1 and 2). **Supplementary Figure 3.** Change in how acceptable participants found elements of risk-stratified bowel cancer FIT thresholds and screening intervals (juries 3 and 4). **Supplementary Figure 4.** Participants’ evaluation (collected in questionnaire 2).

## Data Availability

The data that support the findings of this study are available via the University of Cambridge Data Repository (https://doi.org/10.17863/CAM.90816). Formal requests to PCU_DATA@medschl.cam.ac.uk for access to pseudo-anonymised transcripts will be considered via a data-sharing agreement that indicates the criteria for data access and conditions for research use and will incorporate privacy and confidentiality standards to ensure data security. The study protocol, jury outlines, participant information pack, consent forms, questionnaires, and topic guides are available on the repository. Data will be available upon publication with no end date as indicated.

## Abbreviations

AUC: Area under the receiver operator curve
BC: Bowel cancer
BMI: Body mass index
FIT: Faecal immunochemical testing
PPI: Patient and public involvement
