# Supplementary material for: Implementation of risk stratification within bowel cancer screening: a community jury study exploring public acceptability and communication needs
Source: BMC Public Health. 2023 Sep 15;23:1798. doi: 10.1186/s12889-023-16704-6 (PMC10503141; doi:10.1186/s12889-023-16704-6)
Supplement: Supplementary file 1 — Additional file 1: Supplementary Table 1. Overview of the expert presentations included in the community juries. Supplementary Table 2. Thematic analysis coding frame. Supplementary Table 3. Pre-jury questionnaire (juries 1 and 2). Supplementary Table 4. Post-jury questionnaire (juries 1 and 2). Supplementary Table 5. Pre-jury questionnaire (juries 3 and 4). Supplementary Table 6. Post-jury questionnaire (juries 3 and 4). Supplementary Figure 1. Participants’ beliefs about cancer (collected in questionnaire 1). Supplementary Figure 2. Change in how reasonable or comfortable participants were with elements of risk-stratified bowel cancer screening eligibility (juries 1 and 2). Supplementary Figure 3. Change in how acceptable participants found elements of risk-stratified bowel cancer FIT thresholds and screening intervals (juries 3 and 4). Supplementary Figure 4. Participants’ evaluation (collected in questionnaire 2). [file 12889_2023_16704_MOESM1_ESM.docx]

Implementation of risk stratification within
bowel cancer screening:

A community jury study exploring public acceptability and communication needs

**Supplementary Table 1. Overview of the expert presentations included in the community juries.**

| **Jury 1 and 2** | | **Jury 3 and 4** | |
| --- | --- | --- | --- |
| Day 1 | | | |
| 1. Why do we screen for bowel cancer and what are the potential benefits and harms – General Practitioner and Professor of General Practice | - Definition of screening - Bowel cancer statistics and screening pathway - Potential benefits of bowel screening (including earlier detection, prevention, and reassurance) - Potential harms of bowel screening (including false positives/negatives, physical harm, overtreatment, overdiagnosis) - A key issue for bowel cancer is the limited availability of colonoscopies | 1. Why we screen for bowel cancer and what are the potential benefits and harms – General Practitioner and Professor of General Practice | - Definition of screening - Bowel cancer statistics and screening pathway - Potential benefits of bowel screening (including earlier detection, prevention, and reassurance) - Potential harms of bowel screening (including false positives/negatives, physical harm, overtreatment, overdiagnosis) - A key issue for bowel cancer is the limited availability of colonoscopies - Why are there two steps to bowel cancer screening? - Why is bowel cancer screening offered every two years? |
| 2. Ethical considerations around screening and determining eligibility – Associate Professor of Philosophy of Public Health | - Introduction of core principles in medical ethics (do good, do no harm, treat people fairly and respect choices) - Balancing these principles in the context of screening eligibility | 2. Ethical considerations around bowel cancer screening programmes – Associate Professor of Philosophy of Public Health | - Introduction of core principles in medical ethics (do good, do no harm, treat people fairly and respect choices) - Medical rationing scenarios - Balancing these principles in the context of screening intervals and FIT thresholds |
| 3. Potential effects of introducing risk stratification to determine entry into the bowel cancer screening programme – Research Fellow specializing in public health-related modelling | - Current eligibility for bowel cancer screening - Bowel cancer risk and risk factors - Risk assessment and risk stratification - Potential advantages of risk stratification (including a better balance of benefits and harms, prevention of cancer deaths, reduced cost, better allocation of NHS resources) - Potential disadvantages of risk stratification (including organisational factors, expense, repeat risk assessment, missing cancers in low risk people) | 3. Current approach to bowel cancer screening and how risk stratification could be incorporated (using age, sex, and prior FIT result) – Associate Professor of Public Health | - The current bowel cancer screening programme (including screening intervals and FIT thresholds) - Definition of risk stratification - Age, sex and prior FIT result as risk factors - Potential benefits of using these factors for risk stratification (Including better balance of harms and benefits of screening, reduction in cancers missed, reduction in screening burden) - Potential barriers to risk stratification (including complexity, public understanding and acceptability, potential discrimination) |
| Day 2 | | | |
|  |  | 4. How risk stratification could be incorporated into bowel cancer screening in the future (using lifestyle and/or genetic factors) – Associate Professor of Public Health | - Lifestyle and genetic risk factors for bowel cancer - Risk prediction modelling - Potential additional barriers (including additional data collection, reliability of risk factors and models, how risk translates to screening recommendations) |

**Supplementary Table 2. Thematic analysis coding frame.**

| 1. **Proposed strategy to determine eligibility/thresholds/intervals (verdict)** |
| --- |
| - 1. Using data within system |
| - 1. Collecting additional data |
| - 1. Additional comments |
| 1. **Pros of risk stratification over alternative strategies** |
| - 1. Individual benefit |
| Learn about yourself |
| Logical (given the restraints) |
| Opportunity for more screening if high-risk |
| Opportunity for early detection |
| - 1. NHS and resources use |
| Balance benefits to NHS and public |
| Cost-effective |
| More efficient distribution of resources |
| - 1. Potential to avoid harms |
| Harms are ‘collateral damage’ |
| Harms weren’t important in decision making |
| - 1. Potential for behaviour change |
| - 1. Other |
| 1. **Cons of risk stratification over alternative strategies** |
| - 1. Distasteful or unethical perception |
| - 1. Too complex or expensive for NHS |
| Expensive |
| Better to invest elsewhere e.g. colonoscopies |
| Labour-intensive to collect data |
| Overload the system |
| When to stop |
| - 1. Access to screening in low risk |
| - 1. High risk doesn't mean will get cancer |
| - 1. Other |
| Are FIT results accurate enough to base stratification on? |
| Will high-risk take up screening? |
| Will low-risk have unhealthier lifestyles? |
| 1. **Confusion over risk stratification** |
| Concept of FIT thresholds |
| Family history equals genetics |
| High-risk characteristics would be automatically included |
| Involvement of GP |
| Low-risk characteristics would be completely excluded |
| More people might require screening |
| Not referred despite concerning amount of blood in faeces |
| Risk assessment and screening |
| Risky behaviours would exclude people from screening |
| 1. **Other** |
| - 1. Access to GP and support |
| - 1. Cancer risk worry |
| - 1. Fairness and ethics |
| Fair means all included in risk assessment |
| Fair means choice and respecting individual decisions |
| Including choices as risk factors |
| ‘Rewarding’ unhealthy behaviour |
| - 1. How much benefit is needed |
| - 1. Individuals who cannot provide information |
| - 1. Individuals who don't want to take part |
| - 1. Lifestyle risk factors |
| Balance concerns and risk prediction |
| BMI more objective than behaviours (smoking, diet, alcohol, exercise) directly |
| Cost of changing lifestyle/potential for change |
| Honesty (but that’s for own good) |
| Lifestyle does change |
| People probably know what they should do |
| Right to choose unhealthy behaviours |
| Weighting of lifestyle factors in risk model |
| - 1. Other |
| Risk stratification might be applied to other cancers |
| Young people with lives ahead of them versus older people with higher risk of disease |
| Collecting and securing personal data |
| 1. **Communication** |
| - 1. Channels and outlets |
| - 1. Language |
| - 1. Level of information about individual risk |
| - 1. Level of information about the programme |
| - 1. Level of information provided versus available elsewhere |
| - 1. Prevention advice and education |
| - 1. Programme publicity |
| - 1. Other |
| 1. **Other general points** |
| - 1. Personal stories |
| - 1. Evaluation and feedback |

**Supplementary Table 3. Pre-jury questionnaire (juries 1 and 2).**

**STRAT-BCS:** STRATification in Bowel Cancer Screening – Community jury

**Online questionnaire (1) – to be completed before the community juries**

| Question | Answer | Validation of question/reference |
| --- | --- | --- |
| *[Note that this content will be delivered online so the formatting looked slightly different to in this document. The participants also will not see the headings in grey boxes.]* | | |
| ***Consent*** | | |
| I confirm that I have read and understood the participant information sheet and have given consent to take part in this study. | - [tick box] |  |
| I understand that my taking part is voluntary and that I am free to leave at any time, without giving any reason. | - [tick box] |  |
| I understand that anonymous information collected as part of my involvement in this study may be looked at by responsible individuals from the sponsor, regulatory authorities and research personnel. | - [tick box] |  |
| I understand that information collected as part of my involvement in this study will be used to support other research in the future, and may be shared anonymously with other academic researchers external to the project within the UK and beyond. | - [tick box] |  |
| By clicking 'I agree' you are indicating that you have read and understood this consent form, and agree to participate in this research study.  [‘I agree’/submit form] | | |
| Thank you for agreeing to complete this questionnaire. Please answer every question. If you are uncertain about how to answer a question, then please select the closest option. | | |
| **Demographics** | | |
| ***Demographic information***  *In this first section we would like to ask you a few questions about yourself. These questions allow us to make sure we are including people from a range of different backgrounds and see if different groups of people have different views. We will not be able to identify you from your answers.* | | |
| How old are you? | - 40-44 years - 45-49 years - 50-54 years - 55-59 years - 60-64 years - 65-69 years - 70-74 years - 75-79 years |  |
| What is your sex? (A question about gender identity follows)  Some of the scenarios we will be discussing will consider whether the sex of individuals should influence when they are invited to screening. This would be the sex that individuals are assigned at birth. To help us see if responses differ between people of different sexes, please let us know your sex assigned at birth. | - Female - Male | Cambridge’s The Healthcare Improvement Studies (THIS) Institute guidance released March 2021 |
| Is the gender you identify with the same as your sex registered at birth? | - Yes - No (please enter your gender identity) | Cambridge’s The Healthcare Improvement Studies (THIS) Institute guidance released March 2021 |
| What is your ethnic group?  Choose one option that best describes your ethnic group or background. | - Asian/Asian British - Black/African/Caribbean/Black British - Mixed/Multiple ethnic group - White - Other (please describe) | Ethnic groups are the top-level groups used in the UK Census and PAMCo^1^ |
| What is your highest education level? | - Finished school at or before the age of fifteen - Completed GCSEs, O Levels or equivalent - Completed A Levels or equivalent - Completed further education but not a degree - Completed a Bachelor’s degree - Completed a Master’s degree or PhD - Other (please describe) | Validated from Cancer Awareness Measure (CAM) toolkit, (pre-2014, as 2014 onwards has not been validated)^2^ |
| Which of the following best describes the *main income earner’s* main/most recent job?  This could be you: the main income earner is the person in your household with the largest income.  If the main income earner is retired and has a pension, please answer for their most recent occupation. If the main income earner is not in paid employment but has been out of work for less than 6 months, please answer for their most recent occupation. | - Business owner – responsible for 1-4 employees - Business owner – responsible for 5-24 employees - Business owner – responsible for 25+ employees - Top/senior manager in large organisation; qualified senior professional *e.g. main board director, senior civil servant, headteacher, partner in professional practice, surgeon* - Middle manager or executive in large organisation; senior manager of small organisation; qualified professional (no senior management responsibility) *e.g. department manager, teacher, engineer, accountant, doctor, manager of small building firm* - Non-management office role; middle manager in small organisation; qualified nurse; performing artist *e.g. secretary, personal assistant, clerical worker, office worker, call centre agent, salesperson, nurse or nursery nurse, police constable/sergeant, actor, musician, sportsperson* - Manual worker - manager – responsible for 25+ employees - Manual worker - manager – responsible for 1-24 employees - Skilled manual worker (no responsibility for other employees) *e.g. HGV driver, rain/bus/ambulance driver, chef, hairdresser, mechanic, plumber, bricklayer, carpenter, painter, electrician, caterer, specialised machinery operator, fire-fighter, pub/bar worker* - Unskilled or semi-skilled manual worker (no responsibility for other employees) *e.g. farm worker, cleaner, postal worker, van driver, care worker, waiter, taxi driver, shop assistant, apprentice/trainee in skilled trade* - Casual worker or no regular income - Full time student | The ABC1–C2DE framework is a validated method of determining social grade.^3^ PAMCo uses an updated version of this question^1^ |
| **Lifestyle and screening history** | | |
| ***Lifestyle and screening history***  *The following questions are about your lifestyle and your past screening decisions. Please answer as honestly as you can as this will help us with our analysis.* | | |
| In general, would you say your health is | - Excellent - Very good - Good - Fair - Poor | Validated question from CDC for self-rate health^4^ |
| What is your tobacco smoking status? | - Never smoked cigarettes or cigars - Used to smoke cigarettes or cigars - Smoke up to 20 cigarettes or cigars per day - Smoke 20 or more cigarettes or cigars per day | From lung cancer study, Smits et al., 2018^3^ |
| How would you describe your weight? | - Underweight - About the right weight - Slightly overweight - Very overweight |  |
| Have you ever had cancer? | - Yes - No |  |
| Have your parents or any brothers or sisters ever had cancer? | - Yes - No - Don’t know/prefer not to answer |  |
| Has anyone close to you (e.g. a partner or close friend) ever had cancer? | - Yes - No - Don’t know |  |
| Have you been invited to take part in any of the following screening programmes (please note that they are not offered to everyone)?  Information is in the format of (what it involves, who it is offered to) | - Yes/No/Not Eligible   - Abdominal Aortic Aneurysm screening (ultrasound, men aged over 65)   - Bowel Cancer screening (poo sample or colonoscopy, men and women aged 60-74)   - Breast Cancer screening (mammogram, women aged 50-70)   - Cervical Cancer screening (smear test, women aged 25-64) |  |
| Which of the following screening programmes did you take up the invitation to take part in? | - Yes/No/Not Eligible   - Abdominal Aortic Aneurysm screening (ultrasound, men aged over 65)   - Bowel Cancer screening (poo sample or colonoscopy, men and women aged 60-74)   - Breast Cancer screening (mammogram, women aged 50-70)   - Cervical Cancer screening (smear test, women aged 25-64) |  |
| **Thoughts and beliefs about cancer** | | |
| *The next section asks about your thoughts and beliefs about cancer. Please answer as honestly as you can as this will help us with our analysis. The questions are about YOUR opinion and so there is not a correct answer.* | | |
| Below are some statements that are sometimes made about cancer. For each of these statements how much do you agree or disagree with them?   - normal activities and responsibilities. - Most cancer treatment is worse than the cancer itself. - I would NOT want to know if I have cancer. - Cancer can often be cured. - Going to the doctor as quickly as possible after noticing a symptom of cancer could increase the chances of surviving. - Some people think a diagnosis of cancer is a death sentence. | - Strongly agree - Agree - Neither disagree nor agree - Disagree - Strongly disagree | Cancer beliefs, taken from Smits et al., 2018^3^ (and made specific to bowel cancer, rather than lung cancer), which were based on validated questions from the ABC measure.^5^ |
| **Risk stratification scenarios** | | |
| ***Developing targeted screening programmes by looking at risk factors for bowel cancer***  *Because bowel cancer is quite rare, it would be very expensive to screen all adults in the UK and most of them would not benefit from the screening programme. One way of reducing the cost of screening and increasing the number of people who benefit is to only offer screening to people who we think are more likely to develop bowel cancer.*  *A person’s risk of developing bowel cancer depends on many factors. These include age, sex, smoking, BMI/being overweight, diet, how much physical activity someone does and family history.*  *Please consider the following scenarios and answer the questions honestly. We would like to know YOUR opinion.* | | |
| Age and sex  Imagine how you would feel in the following scenario…  Let’s say you are feeling fine. You receive a letter inviting you to screening for bowel cancer because you have reached a certain age. The letter states that ‘experts recommend that men get their first screening test at age 50 and women at age 60.’ They then explain that ‘the reason they recommend starting earlier in men is because this cancer is more common in men than women’.   1. How reasonable does it seem to you that experts recommend using age and sex to decide when to start screening? 2. How reasonable does it seem to you that, in this scenario, men would be offered screening earlier than women? 3. How comfortable are you with experts using age and sex to decide when you should start screening for bowel cancer? | - Not at all reasonable – extremely reasonable (1-6)   - Not at all reasonable   - Not very reasonable   - Somewhat unreasonable   - Somewhat reasonable   - Very reasonable   - Extremely reasonable - Not at all comfortable – extremely comfortable (1-6) | Adapted from ‘Acceptability and potential impact on uptake of using different risk stratification approaches to determine eligibility for screening: a population based survey’ survey^6^ |
| Phenotypic risk score  Now imagine that you receive a different letter inviting you to screening. In that it describes how experts have developed a calculator to estimate how likely an individual is to develop bowel cancer, and to identify at what age you should first have screening for bowel cancer.  This calculator, which is based on scientific research studies, uses information available from routine GP records, such as your age, sex, BMI (body mass index, a measure that uses your weight and height to work out if your weight is healthy) and smoking status. You are also asked to fill out a questionnaire for the calculator, which asks about your family history of cancer and your lifestyle, including how much physical activity you do and your diet.  Like the screening tests, it is not 100% reliable. Someone who is estimated to be at higher risk based on the calculator is not destined to get bowel cancer. A low risk estimate also does not mean someone is completely without risk of getting bowel cancer.   1. How reasonable does it seem to you that doctors would require you to fill out a questionnaire to predict your risk of bowel cancer? 2. How comfortable would you be filling in the questionnaire and giving information about your family history and lifestyle? 3. How reasonable does it seem to you that experts would use a calculator that includes these factors to predict each person’s risk of bowel cancer and use that to decide what age to start screening for bowel cancer? 4. How reasonable does it seem to you that people at higher risk based on this calculator would be invited to screening for bowel cancer at a younger age than people at lower risk? 5. How comfortable are you with experts using a cancer risk calculator based on these factors to decide what age you should start screening for bowel cancer? 6. How comfortable would you be about having to wait until you were older to start screening for bowel cancer if you were low risk based on this calculator? | - Not at all reasonable – extremely reasonable (1-6) - Not at all comfortable – extremely comfortable (1-6) |  |
| Genetic risk score  Having certain genes can also increase your risk of bowel cancer. Imagine that you are invited to be tested for these high-risk genes by providing a sample of blood or a cheek swab. This information would then be used to calculate how likely you are to develop bowel cancer and used to decide what age to start screening.  The relationship of these genes with the risk of bowel cancer is based on scientific research. Like the risk calculators above, the calculator based on genes is not 100% reliable. Someone who is estimated to be at higher risk based on their genes is not destined to get bowel cancer. A low risk estimate also does not mean someone is completely without risk of getting bowel cancer.   1. How reasonable does it seem to you that doctors would require you to provide a sample of blood or a cheek swab to predict your risk of bowel cancer? 2. How comfortable would you be providing a sample of blood or a cheek swab to allow experts to test whether you have high-risk genes? 3. How reasonable does it seem to you that experts would use a genetic test to predict each person’s risk of bowel cancer and use that to decide what age to start screening for bowel cancer? 4. How reasonable does it seem to you that people at higher risk based on their genes would be invited to screening for bowel cancer at a younger age than people at lower risk? 5. How comfortable are you with doctors using a genetic risk calculator to decide what age you should start screening for bowel cancer? 6. How comfortable would you be about having to wait until you were older to start screening for bowel cancer if you were low risk based on a genetic risk calculator? | - Not at all reasonable – extremely reasonable (1-6) - Not at all comfortable – extremely comfortable (1-6) |  |
| *Thank you for completing the survey. Your time and contribution are very much appreciated.* | | |

References

1 Company PAM. PAMCo Interview and questionnaire. https://pamco.co.uk/how-it-all-works/interview-and-questionnaire/ (accessed 9 Aug 2021).

2 Cancer Research UK, University College London, Kings College London and U of O. Cancer Awareness Measure toolkit version 2.1 Cancer Research UK Cancer Awareness Measure (CAM) Toolkit (version 2.1). 2007. https://www.cancerresearchuk.org/sites/default/files/health_professional_cancer_awareness_measure_toolkit_version_2.1_09.02.11.pdf

3 Smits SE, McCutchan GM, Hanson JA, *et al.* Attitudes towards lung cancer screening in a population sample. *Heal Expect* 2018;**21**:1150–8. doi:10.1111/hex.12819

4 2006 Behavioral Risk Factor Surveillance System Questionnaire. 2006.

5 Simon AE, Forbes LJL, Boniface D, *et al.* An international measure of awareness and beliefs about cancer: development and testing of the ABC. *BMJ Open* 2012;**2**:e001758. doi:10.1136/bmjopen-2012-001758

6 Usher-Smith JA, Harvey-Kelly LLW, Rossi SH, *et al.* Acceptability and potential impact on uptake of using different risk stratification approaches to determine eligibility for screening: A population-based survey. *Heal Expect* 2020;**24**:341–51. doi:10.1111/hex.13175

**Supplementary Table 4. Post-jury questionnaire (juries 1 and 2).**

**STRAT-BCS:** STRATification in Bowel Cancer Screening – Community jury

**Online questionnaire (2) – to be completed at the end of the community jury**

| Question | Answer | Validation of question/reference |
| --- | --- | --- |
| *[Note that this content will be delivered online so the formatting looked slightly different to in this document. The participants also will not see the headings in grey boxes.]* | | |
| Thank you for taking part in the community juries. For the final part of this study, we invite you to complete the follow-up questionnaire below. | | |
| Next | | |
| Thank you for agreeing to complete this questionnaire. Please answer every question. If you are uncertain about how to answer a question, then please select the closest option. | | |
| **Risk stratification scenarios** | | |
| ***Developing targeted screening programmes by looking at risk factors for bowel cancer***  *Because bowel cancer is quite rare, it would be very expensive to screen all adults in the UK and most of them would not benefit from the screening programme. One way of reducing the cost of screening and increasing the number of people who benefit is to only offer screening to people who we think are more likely to develop bowel cancer.*  *A person’s risk of developing bowel cancer depends on many factors. These include age, sex, smoking, BMI/being overweight, diet, how much physical activity someone does and family history.*  *Please consider the following scenarios and answer the questions honestly. We would like to know YOUR opinion.* | | |
| Age and sex  Imagine how you would feel in the following scenario…  Let’s say you are feeling fine. You receive a letter inviting you to screening for bowel cancer because you have reached a certain age. The letter states that ‘experts recommend that men get their first screening test at age 50 and women at age 60.’ They then explain that ‘the reason they recommend starting earlier in men is because this cancer is more common in men than women’.   1. How reasonable does it seem to you that experts recommend using age and sex to decide when to start screening? 2. How reasonable does it seem to you that, in this scenario, men would be offered screening earlier than women? 3. How comfortable are you with experts using age and sex to decide when you should start screening for bowel cancer? | - Not at all reasonable – extremely reasonable (1-6)   - Not at all reasonable   - Not very reasonable   - Somewhat unreasonable   - Somewhat reasonable   - Very reasonable   - Extremely reasonable - Not at all comfortable – extremely comfortable (1-6) | Adapted from ‘Acceptability and potential impact on uptake of using different risk stratification approaches to determine eligibility for screening: a population based survey’ survey^1^ |
| Phenotypic risk score  Now imagine that you receive a different letter inviting you to screening. In that it describes how experts have developed a calculator to estimate how likely an individual is to develop bowel cancer, and to identify at what age you should first have screening for bowel cancer.  This calculator, which is based on scientific research studies, uses information available from routine GP records, such as your age, sex, BMI (body mass index, a measure that uses your weight and height to work out if your weight is healthy) and smoking status. You are also asked to fill out a questionnaire for the calculator, which asks about your family history of cancer and your lifestyle, including how much physical activity you do and your diet.  Like the screening tests, it is not 100% reliable. Someone who is estimated to be at higher risk based on the calculator is not destined to get bowel cancer. A low risk estimate also does not mean someone is completely without risk of getting bowel cancer.   1. How reasonable does it seem to you that doctors would require you to fill out a questionnaire to predict your risk of bowel cancer? 2. How comfortable would you be filling in the questionnaire and giving information about your family history and lifestyle? 3. How reasonable does it seem to you that experts would use a calculator that includes these factors to predict each person’s risk of bowel cancer and use that to decide what age to start screening for bowel cancer? 4. How reasonable does it seem to you that people at higher risk based on this calculator would be invited to screening for bowel cancer at a younger age than people at lower risk? 5. How comfortable are you with experts using a cancer risk calculator based on these factors to decide what age you should start screening for bowel cancer? 6. How comfortable would you be about having to wait until you were older to start screening for bowel cancer if you were low risk based on this calculator? | - Not at all reasonable – extremely reasonable (1-6) - Not at all comfortable – extremely comfortable (1-6) |  |
| Genetic risk score  Having certain genes can also increase your risk of bowel cancer. Imagine that you are invited to be tested for these high-risk genes by providing a sample of blood or a cheek swab. This information would then be used to calculate how likely you are to develop bowel cancer and used to decide what age to start screening.  The relationship of these genes with the risk of bowel cancer is based on scientific research. Like the risk calculators above, the calculator based on genes is not 100% reliable. Someone who is estimated to be at higher risk based on their genes is not destined to get bowel cancer. A low risk estimate also does not mean someone is completely without risk of getting bowel cancer.   1. How reasonable does it seem to you that doctors would require you to provide a sample of blood or a cheek swab to predict your risk of bowel cancer? 2. How comfortable would you be providing a sample of blood or a cheek swab to allow experts to test whether you have high-risk genes? 3. How reasonable does it seem to you that experts would use a genetic test to predict each person’s risk of bowel cancer and use that to decide what age to start screening for bowel cancer? 4. How reasonable does it seem to you that people at higher risk based on their genes would be invited to screening for bowel cancer at a younger age than people at lower risk? 5. How comfortable are you with doctors using a genetic risk calculator to decide what age you should start screening for bowel cancer? 6. How comfortable would you be about having to wait until you were older to start screening for bowel cancer if you were low risk based on a genetic risk calculator? | - Not at all reasonable – extremely reasonable (1-6) - Not at all comfortable – extremely comfortable (1-6) |  |
| **Your experience of the community jury** | | |
| 1. Overall, the presentations by the experts…    1. Helped me understand the issues    2. Were referred to during our deliberation    3. Helped discussion between community jury members | - Strongly disagree - Disagree - Undecided - Agree - Strongly agree - *Additional comments* |  |
| 1. Overall, the facilitator…    1. Helped to stimulate the group’s reflections    2. Respected the opinions of participants | - Strongly disagree - Disagree - Undecided - Agree - Strongly agree - *Additional comments* |  |
| 1. Overall, during the discussions between jurors…    1. I felt comfortable sharing my ideas    2. I could express disagreements    3. I remained engaged throughout the experience    4. Everyone was offered the opportunity to state their views    5. I considered there might be a difference between what I would want for myself, and what I would recommend for the community as a whole    6. I discussed my preferences and values in relation to the jury question    7. I considered the views of others during the deliberation. | - Strongly disagree - Disagree - Undecided - Agree - Strongly agree - *Additional comments* |  |
| 1. The outcomes of our jury…    1. Our community jury reached a clear recommendation/s    2. Our community jury’s recommendation/s directly addressed the question we were given at the start    3. We discussed why we made our recommendations.    4. We made our recommendations for the community as a whole not just for ourselves. | - Strongly disagree - Disagree - Undecided - Agree - Strongly agree - *Additional comments* |  |
| 1. Other comments (OPTIONAL): Do you have anything to add about your experience on this community jury? | - *Additional comments* |  |
| *Thank you for completing the survey. Your time and contribution are very much appreciated.* | | |

References

1 Usher-Smith JA, Harvey-Kelly LLW, Rossi SH, *et al.* Acceptability and potential impact on uptake of using different risk stratification approaches to determine eligibility for screening: A population-based survey. *Heal Expect* 2020;**24**:341–51. doi:10.1111/hex.13175

**Supplementary Table 5. Pre-jury questionnaire (juries 3 and 4).**

**STRAT-BCS II:** STRATification in Bowel Cancer Screening II – Community jury

**Online questionnaire (1) – to be completed before the community juries**

| Question | Answer | Validation of question/reference |
| --- | --- | --- |
| *[Note that this content will be delivered online so the formatting looked slightly different to in this document. The participants also will not see the headings in grey boxes.]* | | |
| ***Consent*** | | |
| I confirm that I have read and understood the participant information sheet and have given consent to take part in this study. | - [tick box] |  |
| I understand that my taking part is voluntary and that I am free to leave at any time, without giving any reason. | - [tick box] |  |
| I understand that anonymous information collected as part of my involvement in this study may be looked at by responsible individuals from the sponsor, regulatory authorities and research personnel. | - [tick box] |  |
| I understand that information collected as part of my involvement in this study will be used to support other research in the future and may be shared anonymously with other academic researchers external to the project within the UK and beyond. | - [tick box] |  |
| By clicking 'I agree' you are indicating that you have read and understood this consent form and agree to participate in this research study.  [‘I agree’/submit form] | | |
| Thank you for agreeing to complete this questionnaire. Please answer every question. If you are uncertain about how to answer a question, then please select the closest option. | | |
| **Demographics** | | |
| ***Demographic information***  *In this first section we would like to ask you a few questions about yourself. These questions allow us to make sure we are including people from a range of different backgrounds and see if different groups of people have different views. We will not be able to identify you from your answers.* | | |
| How old are you? | - 50-54 years - 55-59 years - 60-64 years - 65-69 years - 70-74 years |  |
| What is your sex? (A question about gender identity follows)  Some of the scenarios we will be discussing will consider whether the sex of individuals should influence screening frequency and the threshold of the screening test. This would be the sex that individuals are assigned at birth. To help us see if responses differ between people of different sexes, please let us know your sex assigned at birth. | - Female - Male | Cambridge’s The Healthcare Improvement Studies (THIS) Institute guidance released March 2021 |
| Is the gender you identify with the same as your sex registered at birth? | - Yes - No (please enter your gender identity) | Cambridge’s The Healthcare Improvement Studies (THIS) Institute guidance released March 2021 |
| What is your ethnic group?  Choose one option that best describes your ethnic group or background. | - Asian/Asian British - Black/African/Caribbean/Black British - Mixed/Multiple ethnic group - White - Other (please describe) | Ethnic groups are the top-level groups used in the UK Census and PAMCo^1^ |
| What is your highest education level? | - Finished school at or before the age of fifteen - Completed GCSEs, O Levels or equivalent - Completed A Levels or equivalent - Completed further education but not a degree - Completed a Bachelor’s degree - Completed a Master’s degree or PhD - Other (please describe) | Validated from Cancer Awareness Measure (CAM) toolkit, (pre-2014, as 2014 onwards has not been validated)^2^ |
| Which of the following best describes the *main income earner’s* main/most recent job?  This could be you: the main income earner is the person in your household with the largest income.  If the main income earner is retired and has a pension, please answer for their most recent occupation. If the main income earner is not in paid employment but has been out of work for less than 6 months, please answer for their most recent occupation. | - Business owner – responsible for 1-4 employees - Business owner – responsible for 5-24 employees - Business owner – responsible for 25+ employees - Top/senior manager in large organisation; qualified senior professional *e.g. main board director, senior civil servant, headteacher, partner in professional practice, surgeon* - Middle manager or executive in large organisation; senior manager of small organisation; qualified professional (no senior management responsibility) *e.g. department manager, teacher, engineer, accountant, doctor, manager of small building firm* - Non-management office role; middle manager in small organisation; qualified nurse; performing artist *e.g. secretary, personal assistant, clerical worker, office worker, call centre agent, salesperson, nurse or nursery nurse, police constable/sergeant, actor, musician, sportsperson* - Manual worker - manager – responsible for 25+ employees - Manual worker - manager – responsible for 1-24 employees - Skilled manual worker (no responsibility for other employees) *e.g. HGV driver, rain/bus/ambulance driver, chef, hairdresser, mechanic, plumber, bricklayer, carpenter, painter, electrician, caterer, specialised machinery operator, fire-fighter, pub/bar worker* - Unskilled or semi-skilled manual worker (no responsibility for other employees) *e.g. farm worker, cleaner, postal worker, van driver, care worker, waiter, taxi driver, shop assistant, apprentice/trainee in skilled trade* - Casual worker or no regular income - Full time student | The ABC1–C2DE framework is a validated method of determining social grade.^3^ PAMCo uses an updated version of this question^1^ |
| **Lifestyle and screening history** | | |
| ***Lifestyle and screening history***  *The following questions are about your lifestyle and your past screening decisions. Please answer as honestly as you can as this will help us with our analysis.* | | |
| In general, would you say your health is | - Excellent - Very good - Good - Fair - Poor | Validated question from CDC for self-rate health^4^ |
| What is your tobacco smoking status? | - Never smoked cigarettes or cigars - Used to smoke cigarettes or cigars - Smoke up to 20 cigarettes or cigars per day - Smoke 20 or more cigarettes or cigars per day | From lung cancer study, Smits et al., 2018^3^ |
| How would you describe your weight? | - Underweight - About the right weight - Slightly overweight - Very overweight |  |
| Have you ever had cancer? | - Yes - No |  |
| Have your parents or any brothers or sisters ever had cancer? | - Yes - No - Don’t know/prefer not to answer |  |
| Has anyone close to you (e.g. a partner or close friend) ever had cancer? | - Yes - No - Don’t know |  |
| Have you been invited to take part in any of the following screening programmes (please note that they are not offered to everyone)?  Information is in the format of (what it involves, who it is offered to) | - Yes/No/Not Eligible   - Abdominal Aortic Aneurysm screening (ultrasound, men aged over 65)   - Bowel Cancer screening (poo sample or colonoscopy, men and women aged 60-74)   - Breast Cancer screening (mammogram, women aged 50-70)   - Cervical Cancer screening (smear test, women aged 25-64) |  |
| Which of the following screening programmes did you take up the invitation to take part in? | - Yes/No/Not Eligible   - Abdominal Aortic Aneurysm screening (ultrasound, men aged over 65)   - Bowel Cancer screening (poo sample or colonoscopy, men and women aged 60-74)   - Breast Cancer screening (mammogram, women aged 50-70)   - Cervical Cancer screening (smear test, women aged 25-64) |  |
| **Thoughts and beliefs about cancer** | | |
| *The next section asks about your thoughts and beliefs about cancer. Please answer as honestly as you can as this will help us with our analysis. The questions are about YOUR opinion and so there is not a correct answer.* | | |
| Below are some statements that are sometimes made about cancer. For each of these statements how much do you agree or disagree with them?   - These days, many people with cancer can expect to continue with normal activities and responsibilities. - Most cancer treatment is worse than the cancer itself. - I would NOT want to know if I have cancer. - Cancer can often be cured. - Going to the doctor as quickly as possible after noticing a symptom of cancer could increase the chances of surviving. - Some people think a diagnosis of cancer is a death sentence. | - Strongly agree - Agree - Neither disagree nor agree - Disagree - Strongly disagree | Cancer beliefs, taken from Smits et al., 2018^3^ (and made specific to bowel cancer, rather than lung cancer), which were based on validated questions from the ABC measure.^5^ |
| **Introduction to bowel cancer screening and risk stratification** | | |
| *In the current bowel cancer screening programme in England, participants are asked to complete a test called the faecal immunochemical test (FIT) to see if they need further investigations for bowel cancer. FIT tests check for tiny amounts of blood in poo. A FIT test does not diagnose cancer, but it is a simple way to find out if someone might benefit from further tests. More blood in poo in general means someone is more likely to have a bowel cancer while someone with no blood in their poo is less likely to have a bowel cancer. Like all tests, FIT tests are not perfect. People with a small amount of blood in their poo can still have a cancer and people with lots of blood in their poo do not all have a cancer.*  *In the current programme, FIT results above a certain value are classed as ‘positive’ and those below a certain value are classed as ‘negative’. People who have a ‘positive’ test are referred for a colonoscopy. This is when a thin flexible tube with a camera on the end is used to look inside the bowel. The colonoscopy test does carry some risks. It is usually done in a hospital and requires people to take medicine beforehand to clean out their bowels and they usually need to take a day off work. People are offered a sedative and most people only experience mild pain or no pain during the colonoscopy. Having a colonoscopy is usually safe but in rare cases it can cause harm to the bowel that requires admission to hospital, this happens to about 1 in every 2000 people who have a colonoscopy. Those who test ‘negative’ on FIT are not referred for a colonoscopy but instead invited back to have another FIT test in 2 years.*  *As well as the amount of blood in the poo found on FIT, a person’s risk of developing bowel cancer depends on many other factors. Things that can increase your risk include:*   - *Getting older (8 out of 10 people diagnosed with bowel cancer are over 60)* - *Being male (males are 1.8 times more likely to develop bowel cancer than females)* - *Not being active enough* - *Being overweight* - *Eating a diet high in read and processed meat* - *Eating a diet low in fibre, vegetables and fruits* - *Smoking* - *Drinking too much alcohol* - *Having type 2 diabetes* - *Having inflammatory bowel disease (ulcerative colitis or Crohn’s disease)* - *Having a family history of bowel cancer*   *As the risk of bowel cancer varies based on these factors, we could use this information collected by a questionnaire and the results of the FIT test to determine peoples’ risk of developing bowel cancer. This risk could them be used to determine features of the screening programme. This could mean that instead of inviting everyone to begin bowel cancer screening at the same time, the age at which people are invited to start screening could be based on risk of developing bowel cancer. This could also mean that instead of having a fixed cut-off of blood on the FIT test for everyone, the cut-off for referral to colonoscopy could be based on person’s individual risk. For example, someone who has a higher risk of developing bowel cancer based on their age and sex might be referred for a colonoscopy with a lower FIT result than someone who was at lower risk of developing bowel cancer based on their age and sex. The risk of developing bowel cancer could also be used together with the FIT result to decide how often individual should be invited back for screening. For example, someone who has a higher risk of developing bowel cancer might be invited back for another FIT test more often than someone who was at lower risk of developing bowel cancer.*  *Please answer the following questions honestly. We would like to know YOUR opinion. This exercise may raise questions for you which we hope to address further in the community jury.* | | |
| Acceptability of risk stratification:   1. How acceptable do you think it is to use a person’s individual risk to determine whether they are referred for colonoscopy rather than having a fixed FIT cut-off for everyone? 2. How acceptable do you think it is to use a person’s individual risk to determine when people would be invited back for their next FIT rather than inviting everyone every 2 years? | - Not at all acceptable – extremely acceptable (1-6)   - Not at all acceptable   - Not very acceptable   - Somewhat unacceptable   - Somewhat acceptable   - Very acceptable   - Extremely acceptable | Adapted from ‘Acceptability and potential impact on uptake of using different risk stratification approaches to determine eligibility for screening: a population based survey’ survey^6^ |
| Mode of risk stratification:  All the factors below are known to affect someone’s risk of developing bowel cancer. If elements of the screening programme, like the FIT cut-off or the screening frequency, were going to be determined by peoples’ individual risk, how acceptable do you find using the following factors alongside the FIT result?   1. Sex 2. Age 3. Height and weight 4. Smoking status 5. Family history of cancer 6. Diet 7. Physical activity 8. Genetic information 9. Results of previous screening tests   Now, please rank the options that you considered to be acceptable in order of acceptability, with 1 being most acceptable and 9 being least acceptable. | - Not at all acceptable – extremely acceptable (1-6)   - Not at all acceptable   - Not very acceptable   - Somewhat unacceptable   - Somewhat acceptable   - Very acceptable   - Extremely acceptable - Rank in order of acceptability, with 1 being most acceptable and 9 being least acceptable |  |
| How to decide the cut-off:  If any of the risk factors are used, there are different ways that could be used to decide what the cut-off for referral to have a colonoscopy should be.  How important do you think the following factors are in deciding what the cut-off for a colonoscopy should be?   - The number of cancers missed in people who are not referred for a colonoscopy. - The number of people who have a colonoscopy who do not have a cancer. | - Not at all important – extremely important (1-6)   - Not at all important   - Not very important   - Somewhat unimportant   - Somewhat important   - Very important   - Extremely important |  |
| *Thank you for completing the survey. Your time and contribution are very much appreciated.* | | |

References

1 Company PAM. PAMCo Interview and questionnaire. https://pamco.co.uk/how-it-all-works/interview-and-questionnaire/ (accessed 9 Aug 2021).

2 Cancer Research UK, University College London, Kings College London and U of O. Cancer Awareness Measure toolkit version 2.1 Cancer Research UK Cancer Awareness Measure (CAM) Toolkit (version 2.1). 2007. https://www.cancerresearchuk.org/sites/default/files/health_professional_cancer_awareness_measure_toolkit_version_2.1_09.02.11.pdf

3 Smits SE, McCutchan GM, Hanson JA, *et al.* Attitudes towards lung cancer screening in a population sample. *Heal Expect* 2018;**21**:1150–8. doi:10.1111/hex.12819

4 2006 Behavioral Risk Factor Surveillance System Questionnaire. 2006.

5 Simon AE, Forbes LJL, Boniface D, *et al.* An international measure of awareness and beliefs about cancer: development and testing of the ABC. *BMJ Open* 2012;**2**:e001758. doi:10.1136/bmjopen-2012-001758

6 Usher-Smith JA, Harvey-Kelly LLW, Rossi SH, *et al.* Acceptability and potential impact on uptake of using different risk stratification approaches to determine eligibility for screening: A population-based survey. *Heal Expect* 2020;**24**:341–51. doi:10.1111/hex.13175

**Supplementary Table 6. Post-jury questionnaire (juries 3 and 4).**

**STRAT-BCS II:** STRATification in Bowel Cancer Screening II – Community jury

**Online questionnaire (2) – to be completed at the end of the community jury**

| Question | Answer | Validation of question/reference |
| --- | --- | --- |
| *[Note that this content will be delivered online so the formatting looked slightly different to in this document. The participants also will not see the headings in grey boxes.]* | | |
| Thank you for taking part in the community juries. For the final part of this study, we invite you to complete the follow-up questionnaire below. | | |
| Next | | |
| Thank you for agreeing to complete this questionnaire. Please answer every question. If you are uncertain about how to answer a question, then please select the closest option. | | |
| **Risk stratification questions** | | |
| ***Targeting screening programmes by looking at risk factors for bowel cancer***  *In the current bowel cancer screening programme in England, participants are asked to complete a test called the faecal immunochemical test (FIT) to see if they need further investigations for bowel cancer. FIT tests check for tiny amounts of blood in poo. A FIT test does not diagnose cancer, but it is a simple way to find out if someone might benefit from further tests. More blood in poo in general means someone is more likely to have a bowel cancer while someone with no blood in their poo is less likely to have a bowel cancer. Like all tests, FIT tests are not perfect. People with a small amount of blood in their poo can still have a cancer and people with lots of blood in their poo do not all have a cancer.*  *In the current programme, FIT results above a certain value are classed as ‘positive’ and those below a certain value are classed as ‘negative’. People who have a ‘positive’ test are referred for a colonoscopy. This is when a thin flexible tube with a camera on the end is used to look inside the bowel. The colonoscopy test does carry some risks. It is usually done in a hospital and requires people to take medicine beforehand to clean out their bowels and they usually need to take a day off work. People are offered a sedative and most people only experience mild pain or no pain during the colonoscopy. Having a colonoscopy is usually safe but in rare cases it can cause harm to the bowel that requires admission to hospital, this happens to about 1 in every 2000 people who have a colonoscopy. Those who test ‘negative’ on FIT are not referred for a colonoscopy but instead invited back to have another FIT test in 2 years.*  *As well as the amount of blood in the poo found on FIT, a person’s risk of developing bowel cancer depends on many other factors. These include age, sex, smoking, BMI/being overweight, diet, how much physical activity someone does, family history and genetics. Some or all of these factors can be used alongside the FIT result to estimate a person’s risk of having a bowel cancer. That individual risk could then be used to determine whether they are referred for a colonoscopy or not. This would mean that instead of having a fixed cut-off of blood on the FIT test for everyone, the cut-off for referral to colonoscopy would depend on these other risk factors and the person’s individual risk. For example, someone who has a higher risk of developing bowel cancer based on their age and sex might be referred for a colonoscopy with a lower FIT result than someone who was at lower risk of developing bowel cancer based on their age and sex.*  *Some or all of the risk factors could also be used together with the FIT result to decide when an individual should be invited back for screening. For example, someone who has a higher risk of developing bowel cancer based on their age and sex and FIT result might be invited back for another FIT test sooner than someone who was at lower risk of developing bowel cancer.*  *Please answer the following questions honestly. We would like to know YOUR opinion.* | | |
| Acceptability of risk stratification:   1. How acceptable do you think it is to use a person’s individual risk to determine whether they are referred for colonoscopy rather than a fixed FIT cut-off for everyone? 2. How acceptable do you think it is to use a person’s individual risk to determine when people would be invited back for their next FIT rather than inviting everyone every 2 years? | - Not at all acceptable – extremely acceptable (1-6)   - Not at all acceptable   - Not very acceptable   - Somewhat unacceptable   - Somewhat acceptable   - Very acceptable   - Extremely acceptable | Adapted from ‘Acceptability and potential impact on uptake of using different risk stratification approaches to determine eligibility for screening: a population based survey’ survey^1^ |
| Mode of risk stratification:  All the factors below are known to affect someone’s risk of developing bowel cancer. If elements of the screening programme, like the FIT cut-off or the screening frequency, were going to be determined by peoples’ individual risk, how acceptable do you find using the following factors alongside the FIT result?   1. Sex 2. Age 3. Height and weight 4. Smoking status 5. Family history of cancer 6. Diet 7. Physical activity 8. Genetic information 9. Results of previous screening tests   Now, please rank the options that you considered to be acceptable in order of acceptability, with 1 being most acceptable and 9 being least acceptable. | - Not at all acceptable – extremely acceptable (1-6)   - Not at all acceptable   - Not very acceptable   - Somewhat unacceptable   - Somewhat acceptable   - Very acceptable   - Extremely acceptable - Rank in order of acceptability, with 1 being most acceptable and 9 being least acceptable |  |
| How to decide the cut-off:  If any of the risk factors are used, there are different ways that could be used to decide what the cut-off for referral to have a colonoscopy should be.  How important do you think the following factors are in deciding what the cut-off for a colonoscopy should be?   - The number of cancers missed in people who are not referred for a colonoscopy. - The number of people who have a colonoscopy who do not have a cancer. | - Not at all important – extremely important (1-6)   - Not at all important   - Not very important   - Somewhat unimportant   - Somewhat important   - Very important   - Extremely important |  |
| **Your experience of the community jury** | | |
| 1. Overall, the presentations by the experts…    1. Helped me understand the issues    2. Were referred to during our deliberation    3. Helped discussion between community jury members | - Strongly disagree - Disagree - Undecided - Agree - Strongly agree - *Additional comments* |  |
| 1. Overall, the facilitator…    1. Helped to stimulate the group’s reflections    2. Respected the opinions of participants | - Strongly disagree - Disagree - Undecided - Agree - Strongly agree - *Additional comments* |  |
| 1. Overall, during the discussions between jurors…    1. I felt comfortable sharing my ideas    2. I could express disagreements    3. I remained engaged throughout the experience    4. Everyone was offered the opportunity to state their views    5. I considered there might be a difference between what I would want for myself, and what I would recommend for the community as a whole    6. I discussed my preferences and values in relation to the jury question    7. I considered the views of others during the deliberation. | - Strongly disagree - Disagree - Undecided - Agree - Strongly agree - *Additional comments* |  |
| 1. The outcomes of our jury…    1. Our community jury reached a clear recommendation/s    2. Our community jury’s recommendation/s directly addressed the question we were given at the start    3. We discussed why we made our recommendations.    4. We made our recommendations for the community as a whole not just for ourselves. | - Strongly disagree - Disagree - Undecided - Agree - Strongly agree - *Additional comments* |  |
| 1. Other comments (OPTIONAL): Do you have anything to add about your experience on this community jury? | - *Additional comments* |  |
| *Thank you for completing the survey. Your time and contribution are very much appreciated.* | | |

References

1 Usher-Smith JA, Harvey-Kelly LLW, Rossi SH, *et al.* Acceptability and potential impact on uptake of using different risk stratification approaches to determine eligibility for screening: A population-based survey. *Heal Expect* 2020;**24**:341–51. doi:10.1111/hex.13175

**Supplementary Figure 1. Participants’ beliefs about cancer (collected in questionnaire 1).**
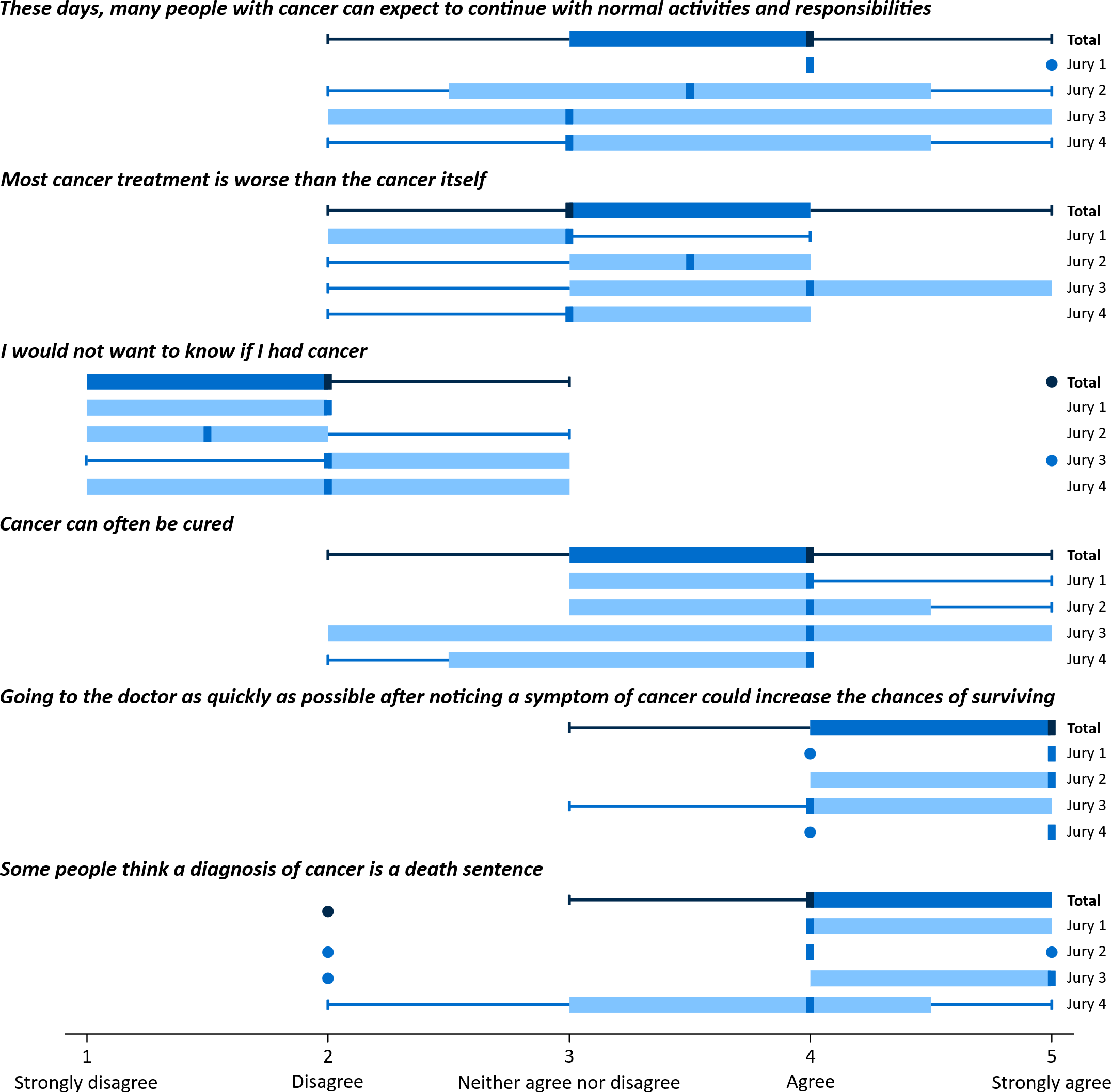


*Total n=30; Jury 1 n=7; Jury 2 n=8; Jury 3 n=7; Jury 4 n=8.*

**Supplementary Figure 2. Change in how reasonable or comfortable participants were with elements of risk-stratified bowel cancer screening eligibility (juries 1 and 2).**


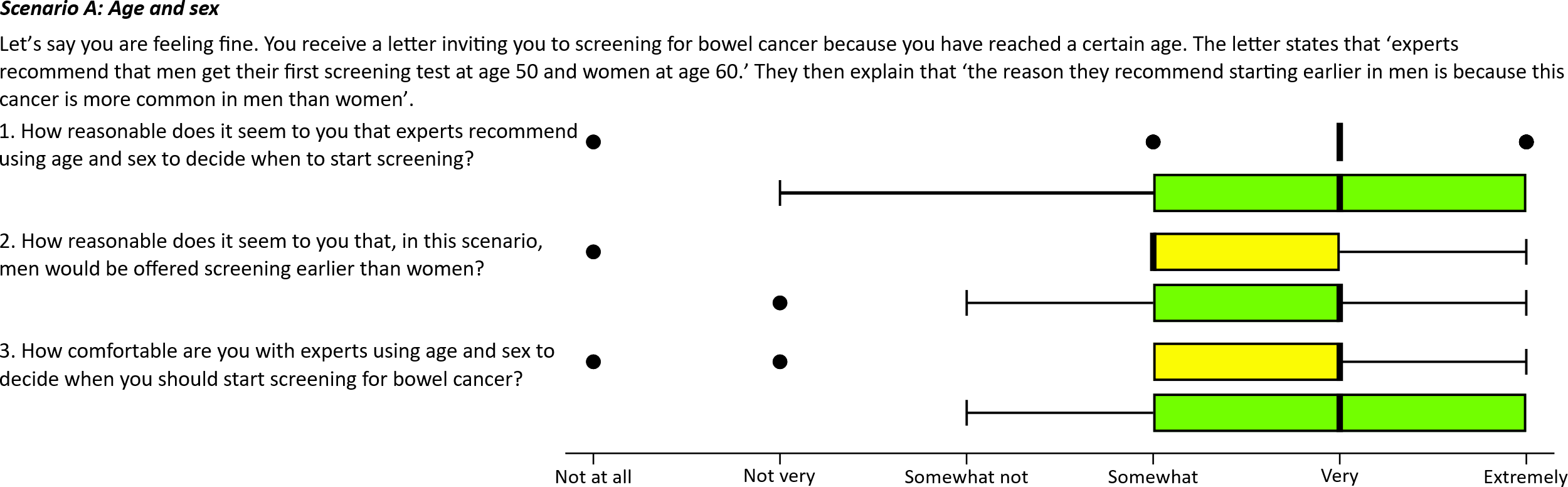


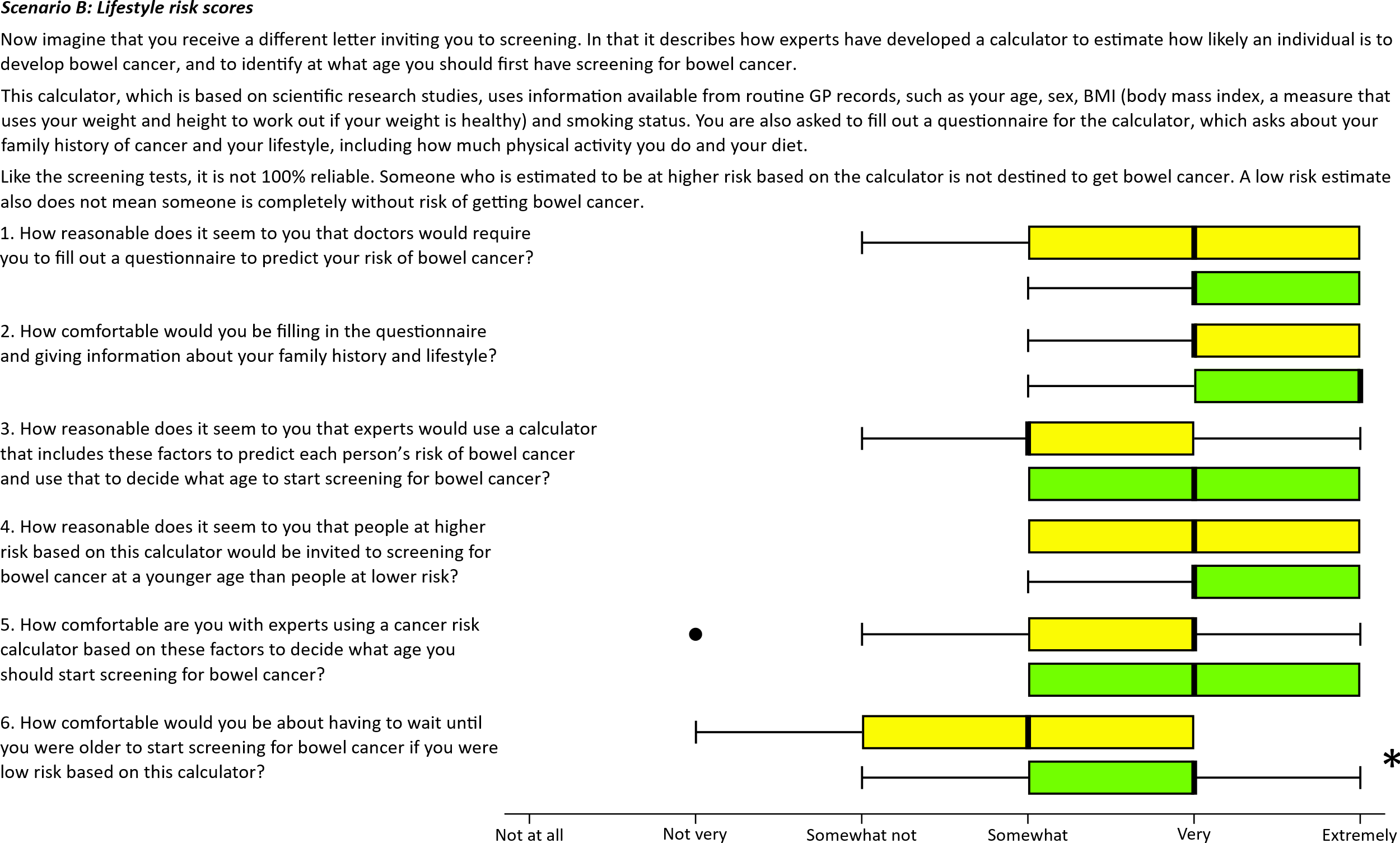


*(Supplementary Figure 2 continued)*


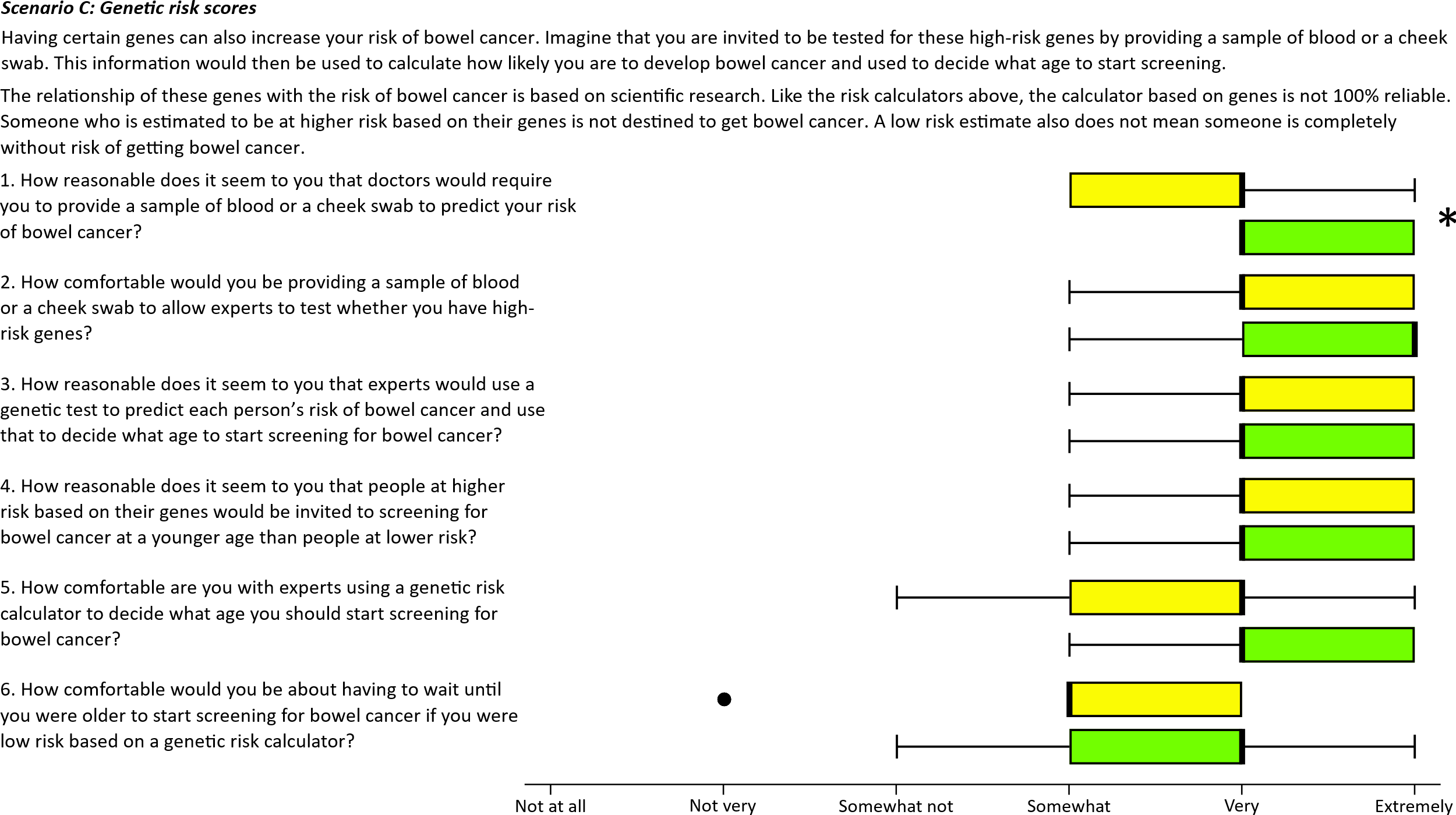


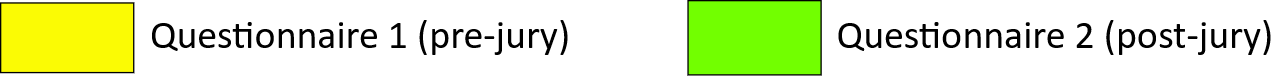


**: p<0.05 in Wilcoxon signed rank test.*

*n=15.*

**Supplementary Figure 3. Change in how acceptable participants found elements of risk-stratified bowel cancer FIT thresholds and screening intervals (juries 3 and 4).**


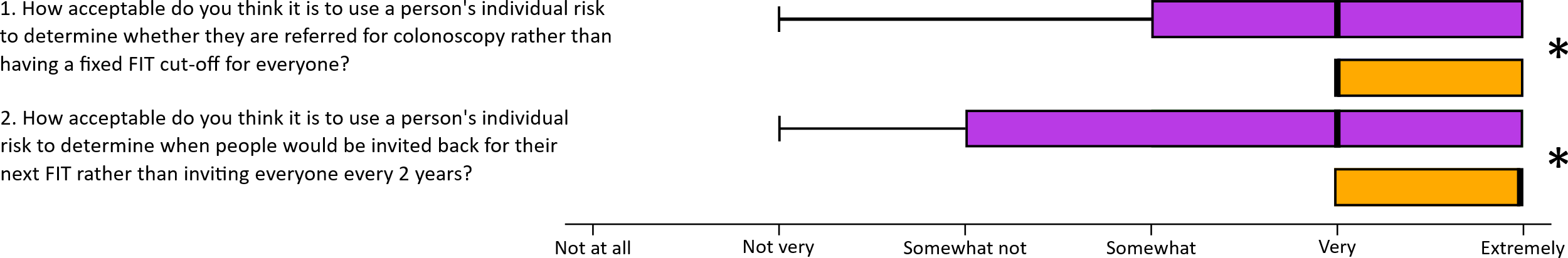


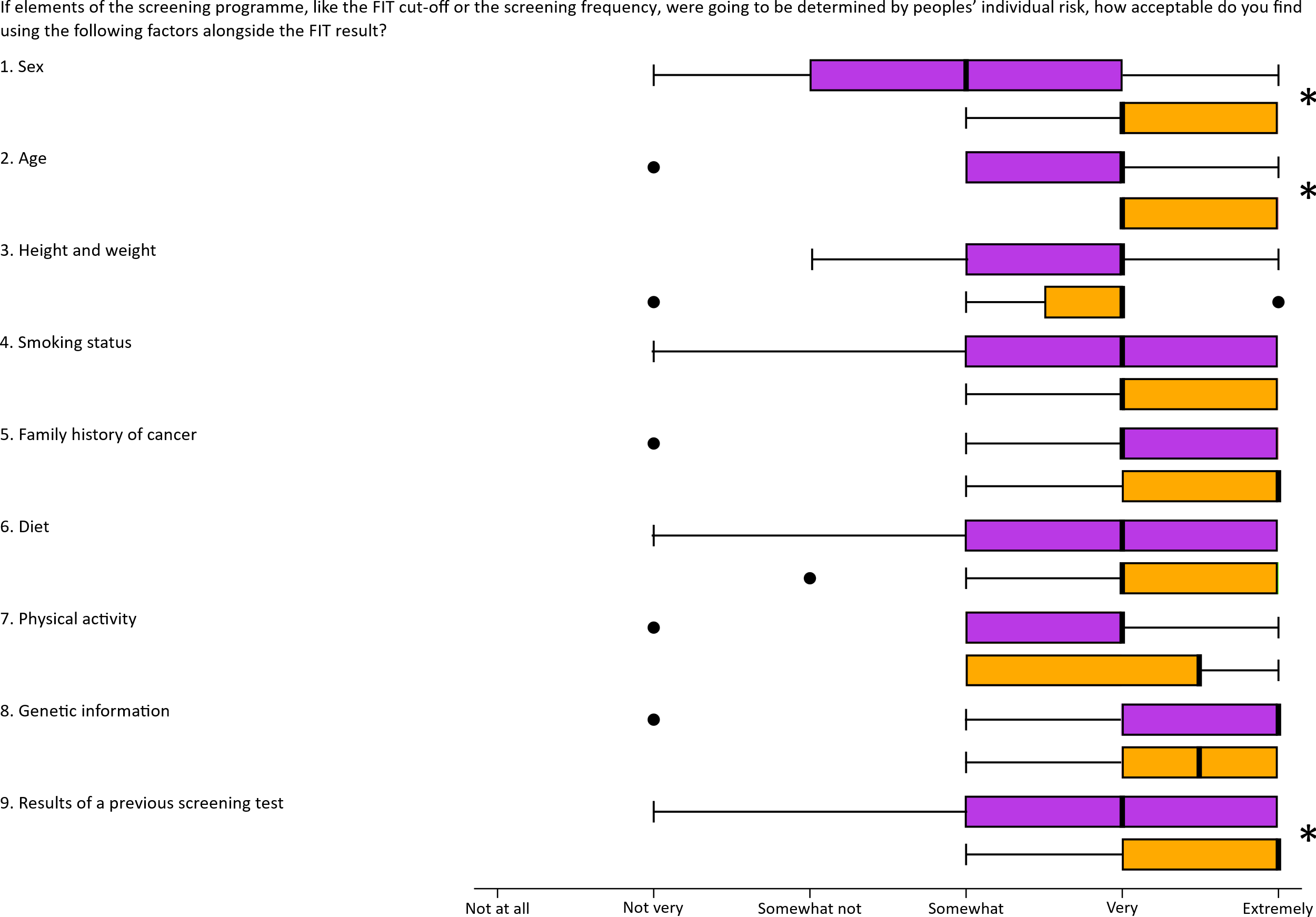


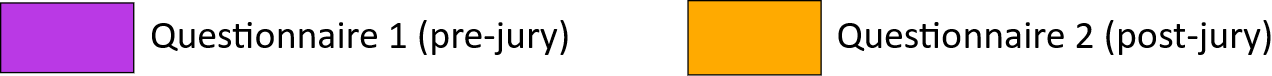


**: p<0.05 in Wilcoxon signed rank test.*

*Questionnaire 1 n=15; Questionnaire 2 n=16.*

**Supplementary Figure 4. Participants’ evaluation (collected in questionnaire 2).**


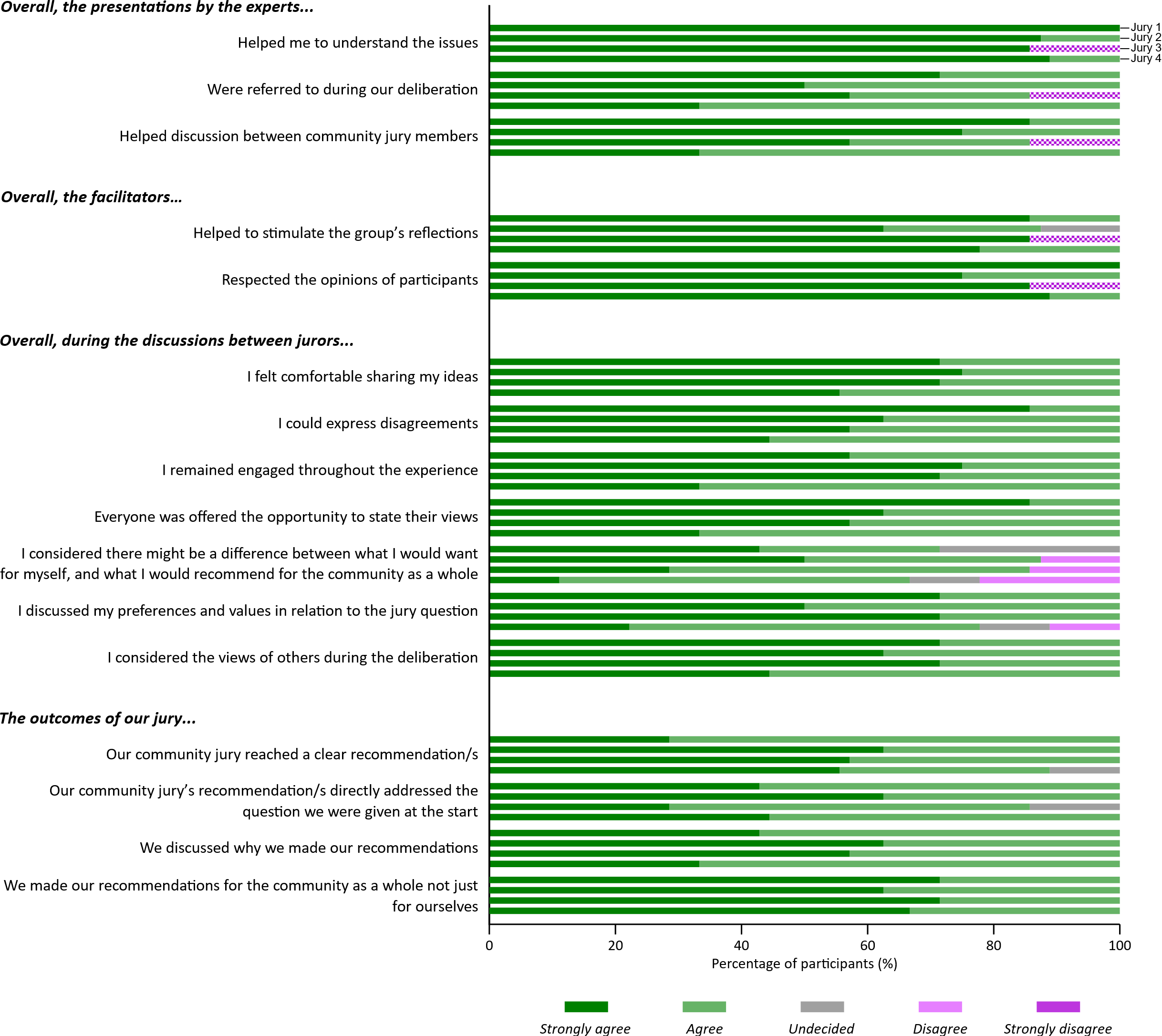


*Note: One participant in jury 3 responded ‘strongly disagree’ to questions 1 and 2 (shaded with cross-hatched) but provided contradictory free-text comments suggesting that they had positive experiences of the presentations and facilitators.*

*Jury 1 n=7; Jury 2 n=8; Jury 3 n=7; Jury 4 n=9.*

**CJ check checklist.**

1. Planning
2.
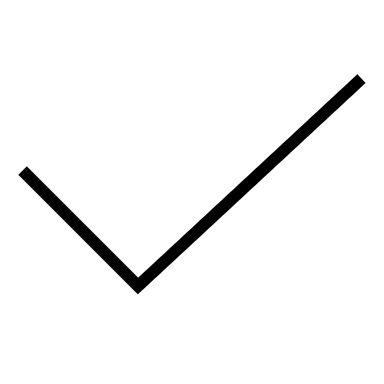
Was the stakeholder/committee’s role clearly described?
3.
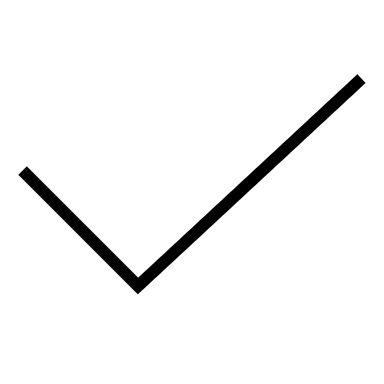
Was the selection of experts (who was chosen and why) adequately described?
4.
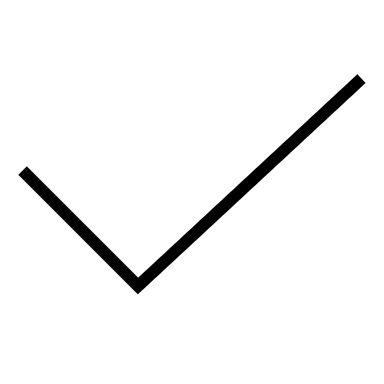
Were the experts roles clearly defined?
5.
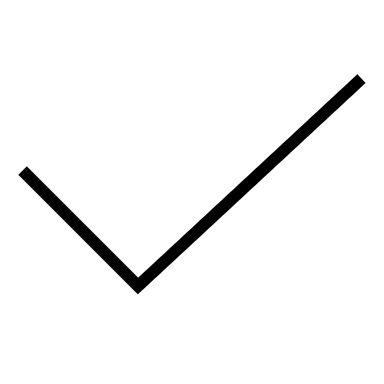
Was the jury ‘charge’ or instruction clearly described?
6. Jurors/participants
7.
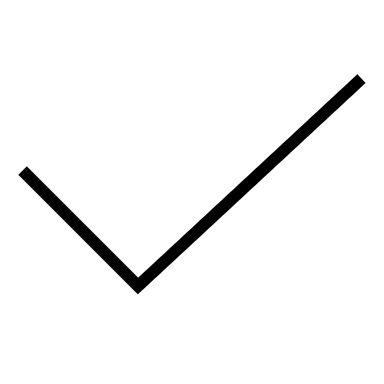

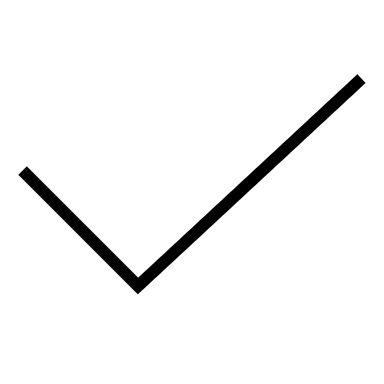
Was the recruitment strategy clearly described?
8. Were inclusion/exclusion criteria reported?
9.
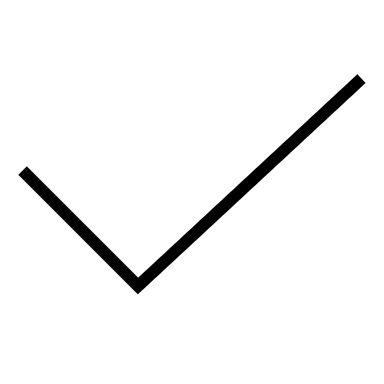
Was the type of participant/juror described? (unaffected public/affected public/advocate)
10.
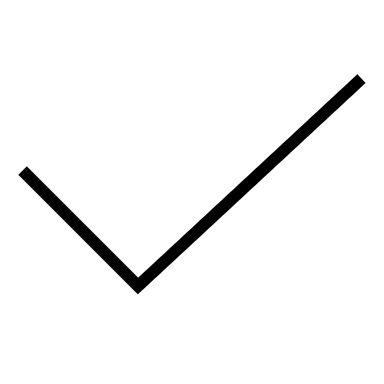
Were the demographics of the jurors reported? (age/gender/education/attainment)
11. Procedure
12.
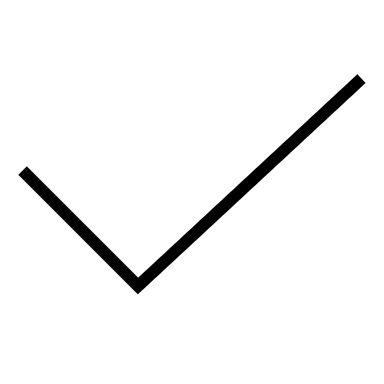
Was the role and experience of the facilitator described (e.g. impartial/informed/member of research team/independent)
13.
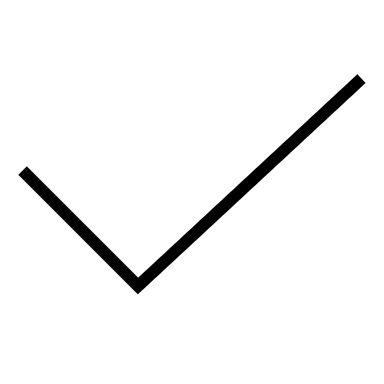
Were materials provided to the jurors adequately described and accessible?
14.
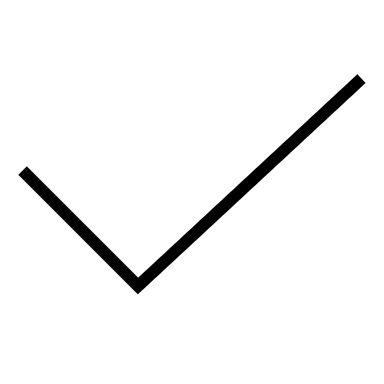

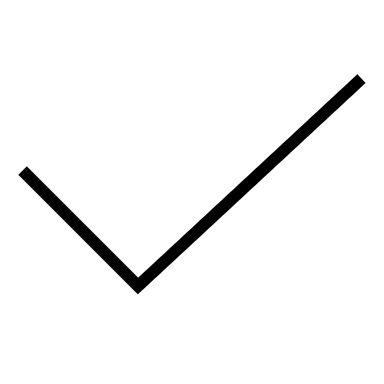
Was the expert cross-examination opportunities described?
15. Was the jury outcome reported?
16. Scheduling
17.
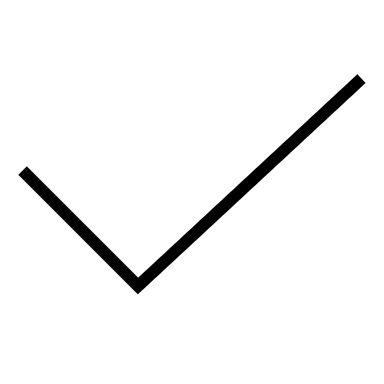
Was the schedule (how often and interval) and length (days/hours) of juror meetings reported?
18.
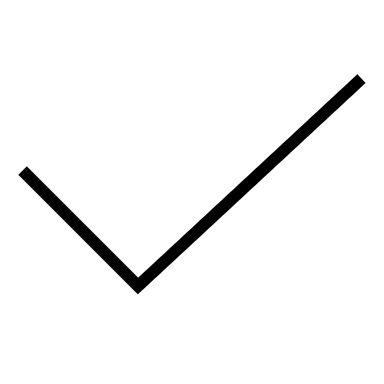
Was the daily schedule of events described?
19.
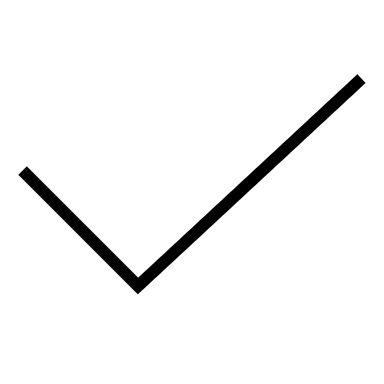
Was the number of presenters and their topics described?
20. Are the expert presentations available? *Presentations are available upon request throught the repository.*
21.
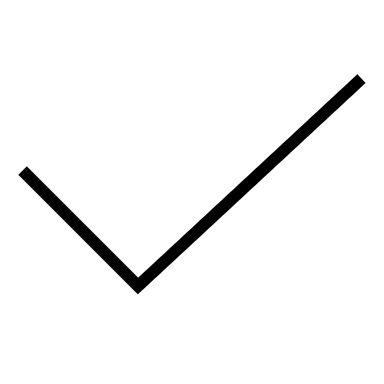
Were the lengths of the presentations reported?
22. Other
23.
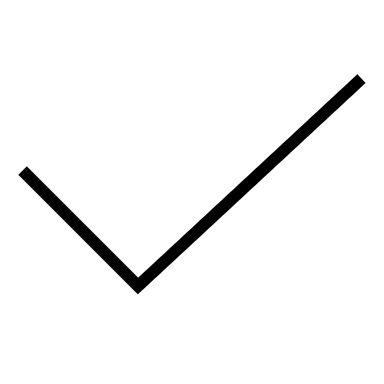
Were the jurors paid?
24.
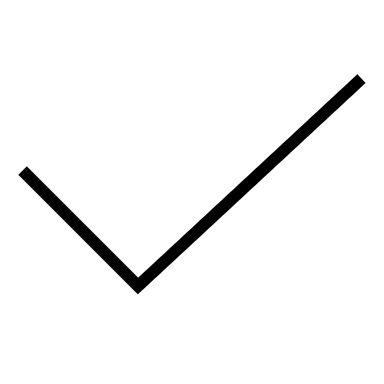
How many jurors were there?
25.
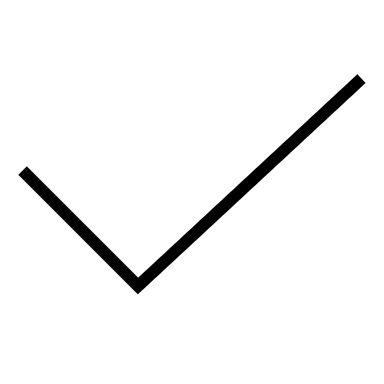
What was the influence of the jury outcome on policy?
26.
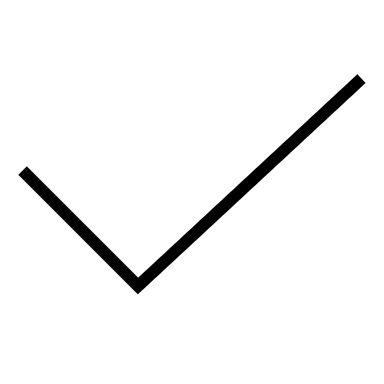
What was the framing/nature of jury deliberations?
27.
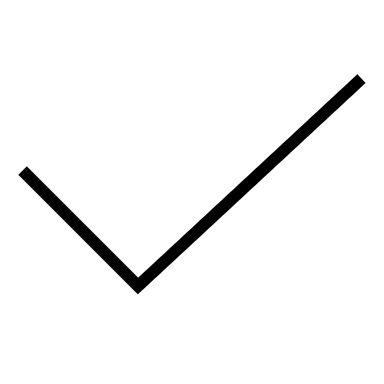
What was the influence of the commissioning body on the jurors?
